# Seasonal influenza vaccination expands hemagglutinin-specific antibody breadth to older and future A/H3N2 viruses

**DOI:** 10.1038/s41541-022-00490-0

**Published:** 2022-06-24

**Authors:** Nina Urke Ertesvåg, Rebecca Jane Cox, Sarah Larteley Lartey, Kristin G-I Mohn, Karl Albert Brokstad, Mai-Chi Trieu

**Affiliations:** 1 Influenza Centre, Department of Clinical Science, University of Bergen, Bergen, Norway; 2 Department of Microbiology, Haukeland University Hospital, Bergen, Norway; 3 Department of Medicine, Haukeland University Hospital, Bergen, Norway

**Keywords:** Inactivated vaccines, Influenza virus, Antibodies, Live attenuated vaccines

## Abstract

History of influenza A/H3N2 exposure, especially childhood infection, shape antibody responses after influenza vaccination and infection, but have not been extensively studied. We investigated the breadth and durability of influenza A/H3N2-specific hemagglutinin-inhibition antibodies after live-attenuated influenza vaccine in children (aged 3-17 years, *n* = 42), and after inactivated influenza vaccine or infection in adults (aged 22-61 years, *n* = 42) using 14 antigenically distinct A/H3N2 viruses circulating from 1968 to 2018. We found that vaccination and infection elicited cross-reactive antibody responses, predominantly directed against newer or future strains. Childhood H3-priming increased the breadth and magnitude of back-boosted A/H3N2-specific antibodies in adults. Broader and more durable A/H3N2-specific antibodies were observed in repeatedly vaccinated adults than in children and previously unvaccinated adults. Our findings suggest that early A/H3N2 exposure and frequent seasonal vaccination could increase the breadth and seropositivity of antibody responses, which may improve vaccine protection against future viruses.

## Introduction

Annual influenza epidemics cause 3-5 million cases of severe illness, and 290,000-650,000 respiratory deaths^1^, with particularly increased mortality in epidemics dominated by influenza A/H3N2 viruses^2^. Since the COVID-19 pandemic, many countries have reinforced strict public-health measures, which also limit the spread of influenza viruses^3^. However, co-infection with influenza viruses in COVID-19 patients has been reported and is associated with increased disease severity and deaths^4,5^. Vaccination is the most effective way to prevent disease and annual influenza vaccination is recommended for high-risk groups^6^. Currently, there are two main types of seasonal influenza vaccines, inactivated influenza vaccine (IIV) and live-attenuated influenza vaccine (LAIV)^7^. Both vaccines aim to induce immunity against the major viral surface glycoprotein, hemagglutinin (HA)^8^. Antibodies directed against the globular head of HA can be measured in the hemagglutinin inhibition assay (HI), where a level of HI ≥40 is an established correlate of protection^9,10^. Influenza A/H3N2 undergoes more rapid viral drift than influenza A/H1N1 and B, facilitating a continual need for seasonal vaccine updates. Since the A/H3N2 virus appeared in 1968, 29 vaccine updates have taken place^11^, versus 15 times for influenza A/H1N1 and 20 times for B^12,13^. Despite frequent vaccine updates, vaccine mismatches due to drifted A/H3N2 viruses during influenza seasons causes low vaccine effectiveness (VE)^14^. Therefore, the correct selection of A/H3N2 strains in seasonal vaccines is critical to improve vaccine-induced protection. However, the vaccine strain selection process has largely ignored the role of human factors, such as pre-existing immunity and repeated annual vaccination, influenced by a lifetime of viral encounters^15^.

There are multiple theories of how pre-existing immunity may impact immune responses. Focus has largely been on how early-life influenza infections and repeated vaccination is shaping the immunity. In 1953 Francis launched his theory of the “original antigenic sin” describing an immunological dominance of the first infecting virus over successive influenza infections^16,17^, where later infecting viruses elicit antibodies against the priming virus. Similarly, *Lesser et al*. found evidence of “antigenic seniority”, where repeated exposure elicited the highest antibodies to “senior” strains from childhood^18^. Antibody cross-reactivity has been modelled to explain an individuals’ complex influenza infection history^19^. Other studies focus on how priming or imprinting with influenza A subtypes in different birth cohorts can preferentially impact the antibody response and potentially reduce influenza mortality^20–24^. A recent theory, termed “back-boosting”, does not restrict cross-reactive antibody responses after recent infection or vaccination to the primary infecting virus, but rather against all previously encountered viruses of the same influenza A subtype^25–28^.

Studies of antibody landscapes against historical and recently circulating viruses are needed to understand how pre-existing immunity and historical exposure affects antibody responses. Furthermore, whether back-boosting responses vary in adults primed with different HAs compared to more naïve children and the effect of repeated vaccination on antibody cross-reactivity are unknown. Our study aimed to provide detailed characteristics of cross-reactive antibody responses in healthy adults and children, using 14 antigenically distinct A/H3N2 viruses which circulated over five decades, from 1968 to 2018. We studied A/H3N2-specific antibodies after recent infection, and single or repeated seasonal vaccination, conducting long-term follow-up^29,30^. We further investigated the extent and maintenance of A/H3N2 HI-antibody “back-boosting”, the impact of “original antigenic sin” and childhood priming. Our findings provide insight to cross-reactive antibody responses by increasing age and repeated vaccination. We show that vaccination elicited cross-reactive antibody responses similarly to infection-induced responses, highlighting the value of annual vaccination.

## Results

**Fig. 1:**
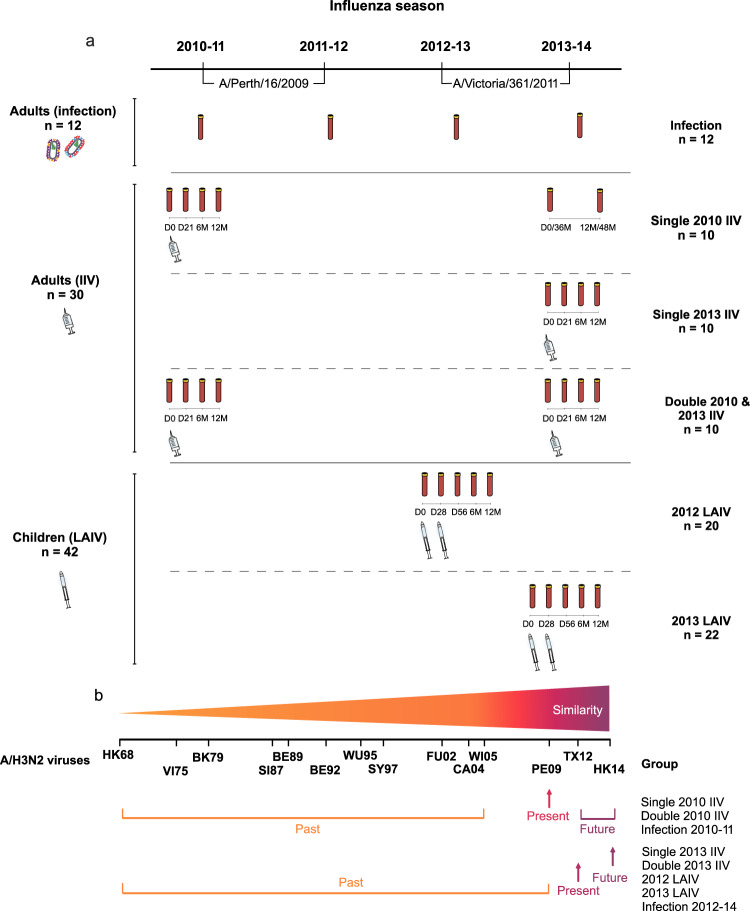
Study design. During the influenza seasons 2010-14, the A/H3N2 component in the seasonal vaccine changed from A/Perth/16/2009 in 2010-12 seasons to A/Victoria/361/2011 in 2012-14 seasons. **a** A group of unvaccinated adults were infected with circulating influenza A/H3N2 viruses, defined as a fourfold seroconversion in HI titres between pre- and post-season blood samples (infection, *n* = 12). Thirty adults were vaccinated with IIV during the study period, generating three different vaccination groups (*n* = 10 each): single 2010 IIV, single 2013 IIV and double 2010 and 2013 IIV. Five individuals from the single 2010 IIV group provided long-term follow-up blood samples 36 and 48 months after vaccination (equivalent to day 0 and 12 months in the 2013-14 season). The children’s cohort were vaccinated with either one (≥9 years old) or two doses (<9 years old) of a live-attenuated influenza vaccine (LAIV) in two different influenza seasons (2012 LAIV (*n* = 20) and 2013 LAIV (*n* = 22)). Blood samples were collected in all vaccinated individuals at day 0 (D0) and post-vaccination day 21/28 (D21/D28), day 56 (D56, only in children), 6 and 12 months (6 M, 12 M). (b) Timeline of A/H3N2 viruses circulated from 1968 to 2018 and the different groups’ perspective of past, present and future viruses according to the timeline. Adults vaccinated or infected in 2010-12 with the A/Perth/16/2009 (PE09) had not yet been exposed to A/Texas/50/2012 (TX12) or A/HongKong/4801/2014 (HK14), defined as future strains for these subjects. Vaccinated or infected adults, and vaccinated children, in 2012-14 had not been exposed to the future strain HK14 which circulated from 2015-18.

We investigated the breadth and durability of influenza A/H3N2-specific antibodies using 14 antigenically distinct A/H3N2 viruses circulating from 1968 to 2018 in groups of adults (vaccinated or infected) and vaccinated children (Fig. 1, Table 1). Vaccinated adults (aged 22-61 years, *n* = 30) received IIV either in 2010, 2013 or both years. Children (aged 3-17 years, *n* = 42) were vaccinated with LAIV in 2012 or 2013. Blood samples were collected in all vaccinated individuals at day 0 and postvaccination day 21/28, day 56 (only children), 6 and 12 months. The unvaccinated adults provided blood samples in September/October each year in 2010-2014, and natural infection was confirmed by seroconversion in twelve adults. The majority of all adults (32/42) and half of children (20/42) were female. No significant differences in age or sex between the infected or vaccinated adult groups or between groups of children receiving LAIV were found (Supplementary Table 1).

**Table 1:** The 14 genetically and antigenically distinct influenza A/H3N2 viruses used in the study.

Influenza season	A/H3N2 vaccine strain^1^	Strains used in study	Abbreviation
1970-72	A/Hong Kong/1/1968-like	A/Hong Kong/1/1968	HK68
1976-80	A/Victoria/3/1975-like	A/Texas/1/1977	VI75
1980-83	A/Bangkok/01/1979-like	A/Bangkok/01/1979	BK79
1988-89	A/Sichuan/02/1987-like	A/Sichuan/02/1987	SI87
1991-93	A/Beijing/353/1989-like	A/Beijing/353/1989	BE89
1993-94	A/Beijing/32/1992-like	A/Beijing/32/1992	BE92
1996-98	A/Wuhan/359/1995-like	A/Wuhan/359/1995	WU95
1998–2000	A/Sydney/5/1997-like	A/Sydney/5/1997	SY97
2004-05	A/Fujian/411/2002-like	A/Wyoming/3/2003	FU02
2005-06	A/California/7/2004-like	A/New York/55/2004	CA04
2006-08	A/Wisconsin/67/2005-like	A/Hiroshima/52/2005	WI05
2010-12	A/Perth/16/2009-like	A/Wisconsin/15/2009	PE09
2012-15	A/Victoria/361/2011-like	A/Texas/50/2012	TX12
2016-18	A/Hong Kong/4801/2014-like	A/Hong Kong/4801/2014	HK14

^1^WHO recommended

The influenza A/H3N2 virus that caused the 1968 pandemic and the 13 predominant circulating A/H3N2 strains until 2018 were selected. All strains were recommended by the World Health Organization (WHO) to be included in seasonal influenza vaccines for the Northern hemisphere. All 14 viruses were egg-grown and inactivated before use in the hemagglutination inhibition assay.

### Infection and vaccination boosted homologous antibodies

**Fig. 2:**
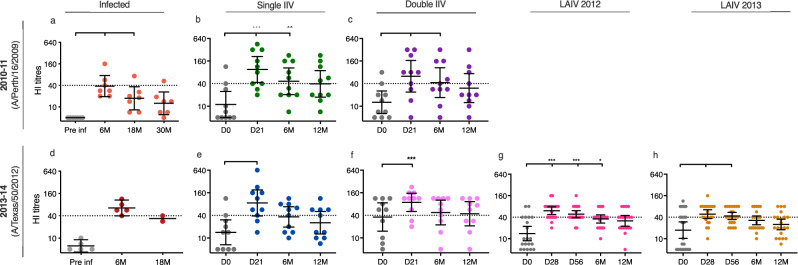
Homologous hemagglutination inhibition (HI) titres after influenza A/H3N2 infection and vaccination in adults and children. Adults were either infected or vaccinated with seasonal inactivated influenza vaccines (IIVs) and children were vaccinated with seasonal live-attenuated influenza vaccines (LAIV). Serum samples were collected once a year before the start of influenza season in infected adults (September/October) and time post-infection was calculated from the season seroconversion occurred. HI titres against homologous A/Perth/16/2009 (H3N2) or A/Texas/50/2012 (H3N2) are shown in adults pre- and post-infection (*n* = 7 or 5, respectively) (**a**, **b**) and in adults pre- (day 0 (DO)) and post-IIV (day 21 (D21), 6 (6 M) and 12 months (12 M)) (*n* = 10 each) (**c–f**). LAIV was not licensed in Europe and Norway until 2012, therefore we do not have vaccination responses against A/Perth/16/2009 in children. HI responses to the homologous A/Texas/50/2012 (H3N2) vaccine strain pre- (day 0) and post-LAIV (day 21, 56, 6, and 12 months) in 2012 (*n* = 20) (**g**) and 2013 (*n* = 22) (**h**). Each symbol represents an individual HI titre. The horizontal lines show geometric mean HI titres with 95% confidence interval. The dotted line indicates an HI titre of 40. Pre- and post-vaccination HI titres were compared using nonparametric repeated measure Friedman test with Dunn’s multiple comparison correction for each vaccination group, except the TX12 infected group due to missing sample for long-term follow-up, which was analysed using non-parametric Wilcoxon matched-pairs signed-rank test for pre- and 6 M post-infection time points. *P < 0.05, **P < 0.01, ***P = < 0.001.

We first tested the homologous HI antibody response after infection or vaccination in adults and children. Twelve unvaccinated adults, with no or low pre-existing antibody titres, seroconverted against influenza A/H3N2 viruses during the 4-year period, probably due to infection. These adults had increases in HI titres after infection, 7 individuals against the A/Perth/16/2009 (PE09) virus (*p* < 0.001) between 2010-12, and 5 adults against the A/Texas/50/2012 (TX12) (*p* = 0.062) in 2012-14 (Fig. 2a, d). Antibody titres then waned overtime in the infected individuals.

We found significant increases in HI titres against the homologous vaccine strains after vaccination in all groups (Fig. 2b, c, e–h). Post-vaccination responses were surprisingly comparable to post-infection responses against PE09 and TX12. A higher number of children had pre-vaccination HI titres ≥40 in the LAIV group vaccinated in 2013 than in 2012, however, post-vaccination titres were similar between the two groups (Fig. 2g, h). The double IIV group had higher pre-vaccination titres (*p* = 0.0637) but comparable post-vaccination titres to the single 2013 IIV group (Fig. 2e, f). This double IIV group also had higher titres than both groups of children pre-vaccination (2012 *p* = 0.039, 2013 *p* = 0.085) and 21/28 days post-vaccination (2012 *p* = 0.067, 2013 *p* = 0.0164) (Fig. 2f, g, h). Notably, only the double IIV group had durable HI titres ≥40 at 12 months post-vaccination while neither the single IIV nor the LAIV children achieved this.

### Vaccination and infection increased heterologous antibodies

**Fig. 3:**
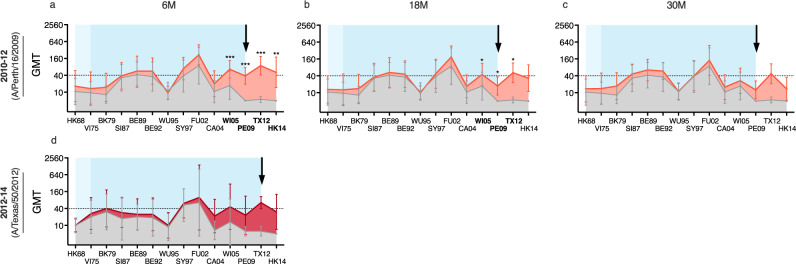
Hemagglutination inhibition (HI) antibody landscapes after A/H3N2 infection in adults. Antibody landscapes were generated with geometric mean HI titres (GMTs) against 14 historical and future influenza A/H3N2 viruses (see Table 1 for the strains used). Error bars represent the 95% confidence intervals of the GMTs. In Norway, the influenza season usually starts at the end of October and peaks between December and March. Blood samples were collected annually in September/October from 2010 to 2014 from unvaccinated adults. Seroconversion (four-fold increase in HI titres) between two time points against the predominantly circulating A/H3N2 viruses were defined as infection. The time post-infection was calculated from the season seroconversion occurred. All individual landscapes are shown in Supplementary Figure 2a. HI antibody landscapes are shown pre-infection (grey) and at 6-9 months (6 M) (**a**), 18–21 months (18 M) (**b**) and 30-33 months (30 M) (**c**) post-infection with PE09, *n* = 7, and at 6-9 months post-infection with TX12, *n* = 5 (**d**). No landscape was generated at 18-21 months post-TX12 infection because only 2 individuals provided serum samples. The viral exposure period is shown in blue based on the group’s median age, and in light blue indicating the oldest individual in the group. Pre- and post-infection HI titres were compared using non-parametric repeated measure Friedman test with Dunn’s multiple comparison correction for each infected group. *P < 0.05, **P < 0.01, ***P = < 0.001.

**Fig. 4:**
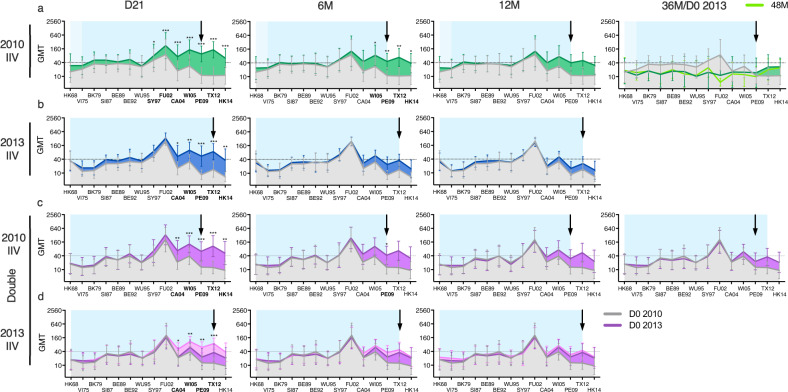
The A/H3N2-specific hemagglutination inhibition (HI) antibody landscapes after vaccination with inactivated influenza vaccines (IIV) in adults. Antibody landscapes were generated using the groups’ geometric mean HI titre (GMT) against 14 antigenically distinct influenza A/H3N2 viruses (see Table 1 for the strains used). Error bars represent the 95% confidence intervals of the GMTs. The pre-vaccination GMTs at day (D)0 are displayed in grey. **a** Antibody landscapes of adults vaccinated with single IIV in 2010, *n* = 10 (single 2010 IIV) at D21, 6 months (M), 12 M, 36 M (equivalent to D0 in 2013) (dark green), and 48 M (equivalent to 12 M in 2013) (light green) post-IIV. **b** Antibody landscapes of adults vaccinated in 2013, *n* = 10 (single 2013 IIV) at D21, 6 M and 12 M post-IIV (dark blue). (**c–d**): Antibody landscapes of adults vaccinated in both 2010 and 2013 (double IIV), *n* = 10, at D21, 6 M, 12 M and 36 M after 2010 IIV (dark purple) (**c**) or after 2013 IIV (light purple) (**d**). The individual vaccinee landscapes can be found in Supplementary Figure 2b–d. The black arrow indicates the vaccination virus, and the dotted line indicates the HI titre of 40. The viral exposure period is shown in blue based on the group’s median age, and light blue indicating the oldest individual in the group. Pre- and post-IIV titres were compared using nonparametric repeated measure Friedman test with Dunn’s multiple comparison correction for each vaccination group. *P < 0.05, **P < 0.01, ***P = < 0.001.

**Fig. 5:**
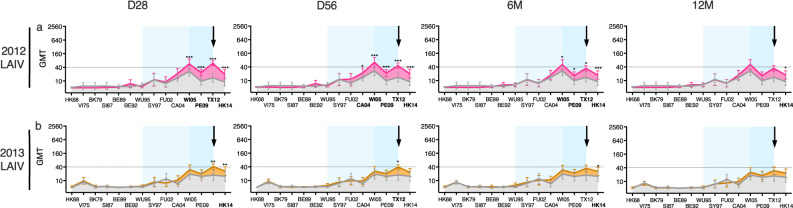
The A/H3N2-specific hemagglutination inhibition (HI) antibody landscapes after vaccination with live-attenuated influenza vaccines (LAIV) in children. Antibody landscapes were generated using the groups’ geometric mean HI titre (GMT) against 14 antigenically distinct influenza A/H3N2 viruses (see Table 1 for the strains used). Error bars represent the 95% confidence intervals of the GMTs. The pre-vaccination GMTs at day (D)0 are displayed in grey. Antibody landscapes of children vaccinated with LAIV in 2012 (2012 LAIV, *n* = 20) (pink) (**a**) or in 2013 (2013 LAIV, *n* = 22) (brown) (**b**) at D28, D56, 6 months (M) and 12 M post-LAIV are shown. Individual landscapes can be found in Supplementary Figure 3. The black arrow indicates the vaccination virus, and the dotted line indicates the HI titre of 40. The viral exposure period is shown in blue based on the group’s median age, and light blue indicating the oldest individual in the group. Pre- and post-LAIV titres were compared using non-parametric repeated measure Friedman test with Dunn’s multiple comparison correction for each vaccination group. *P < 0.05, **P < 0.01, ***P = < 0.001.

To investigate the breadth of antibody responses after infection and vaccination, we evaluated HI antibodies against 14 antigenically distinct A/H3N2 viruses spanning from 1968 to 2018 (Fig. 1b, Table 1). Antibody landscapes were generated from the HI titres pre- and post-exposure for each individual (Supplementary figure 2,3) and from the geometric mean titres (GMTs) for each group (Figs. 3–5).

We observed that infected adults had detectable pre-existing antibodies (>10) against a number of past A/H3N2 viruses, but not against the infecting or future viruses circulating in subsequent years (Fig. 3a, d). Following PE09 infection (n = 7), HI antibodies increased significantly (*p* < 0.01) against the infecting (PE09) and future (TX12, HK14) viruses and back-boosted against the closest virus, WI05, while antibodies against the distant historical viruses were maintained above the pre-infection levels (Fig. 3a). HI titres waned over the next two years post-PE09 infection, but remained elevated above pre-infection levels (Fig. 3b, c). A similar trend of antibody responses was observed after infection with TX12, although not statistically significant probably due to a lower number of subjects in this subgroup (*n* = 5) (Fig. 3d).

The IIV adults also had pre-existing antibodies against historical viruses dating back to HK68 (Fig. 4). Unlike the infection group, all IIV groups had pre-vaccination titres against PE09 and TX12. Vaccination elicited significant increases in HI titres ≥40 at day 21 to the vaccine viruses, the previously circulating (back to SY97 or CA04) and the future strain(s) (TX12 and/or HK14) (Fig. 4a–d). In all IIV groups, the GMTs remained above baseline at 12 months post-vaccination against the four strains WI05, PE09, TX12 and HK14.

The LAIV children had pre-vaccination antibodies against recent (SY97-TX12) and future (HK14) viruses, but no antibodies against historical viruses (HK68-WU95) that circulated before they were born (Fig. 5a, b). A significant increase in HI titres at 28 days post-LAIV was observed against the four strains WI05, PE09, TX12 and HK14 in the 2012 LAIV group (*p* < 0.001) and against TX12 and HK14 in the 2013 LAIV group (*p* < 0.01). These vaccine-induced antibodies waned throughout the 12 months post-LAIV, but remained above pre-vaccination levels. The antibody landscape in children was lower in magnitude and breadth after LAIV compared to after IIV in adults, with HI titres ≥40 post-LAIV only elicited against the TX12 vaccine virus in both groups and additionally WI05 in the 2012 LAIV group.

### Repeated vaccination in adults maintained cross-reactive antibody responses

We followed vaccinees in the single 2010 IIV group (*n* = 5) up to 48 months and observed a decrease in antibody titres to below baseline against most viruses at 36-48 months after vaccination (Fig. 4a, Supplementary Figure 4). In contrast, the double 2010 and 2013 IIV group (*n* = 10) maintained antibodies at or above baseline levels against all 14 viruses at 36 months after 2010 vaccination (first IIV) (Fig. 4c). HI antibodies against these 4 strains WI05-HK14 were boosted following the second IIV in 2013 (*p* < 0.01, except HK14), and remained elevated above the pre-2010 IIV baseline at the end of the 2013 season (Fig. 4a, d). This suggests an advantage of repeated vaccination in inducing durable cross-reactive antibody responses.

### Priming influenced antibody landscapes

**Fig. 6:**
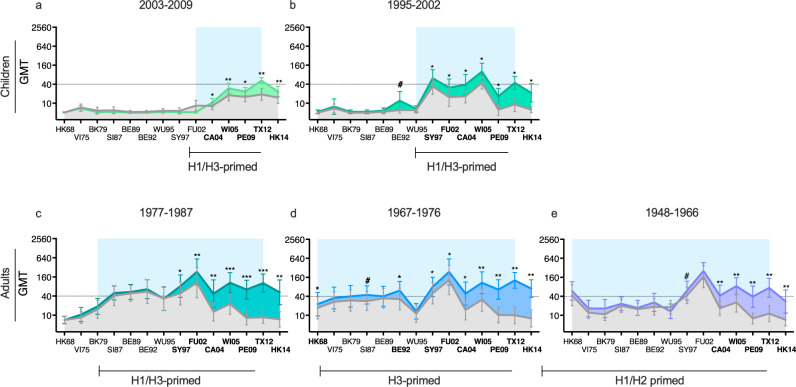
The A/H3N2-specific hemagglutination inhibition (HI) antibody landscapes by birth-year in adults and children. Antibody landscapes were generated using the groups’ geometric mean HI titre (GMT) against 14 antigenically distinct influenza A/H3N2 viruses (see Table 1 for the strains used). Error bars represent the 95% confidence intervals of the GMTs. Children and adults were grouped by their birth year regardless of the year they were vaccinated or infected. The pre-existing HI titres are displayed in grey. Peak titres after vaccination or infection were used (21/28 days post-vaccination or 6 months post-infection). Antibody landscapes post-vaccination of children born between 2003 and 2009 (*n* = 31) in light green (**a**) and children born between 1995 and 2002 (*n* = 11) in dark green (**b**). Antibody landscapes post-vaccination or infection of adults born 1977-1987 (*n* = 14) in ocean green (**c**), adults born 1967-1976 (*n* = 14) in blue (**d**), and adults born 1948-1966 (*n* = 14) in lavender (**e**). The dotted line indicates the HI titre of 40 and the period of viral exposure is highlighted by a light blue background. Pre- and post-vaccination or infection HI titres were compared using non-parametric Wilcoxon matched-pairs signed-rank test with individual ranks computed for each comparison, and Holm-Šídák method for multiple comparisons. ^#^*P* < 0.1, *P < 0.05, **P < 0.01, ***P = < 0.001.

To better understand the effect of priming and age on antibody cross-reactivity after infection and vaccination, we divided children into two birth cohorts based on the recommendation of one or two doses of LAIV; 2003-2009 (3-9 years old, *n* = 31) and 1995-2002 (10-17 years old, *n* = 11). Adults were divided into three birth cohorts according to the likelihood of priming with different influenza A subtypes that circulated when they were born^22^: 1948-1966 (H1/H2 primed, *n* = 14), 1967-1976 (H3 primed, *n* = 14) and 1977-1987 (H1/H3 primed, *n* = 14) (Fig. 6). As expected, the antibody landscapes reflect the lifetime experience of A/H3N2 virus encounters. The children had pre-existing antibodies against recent viruses that circulated in the years since they were born (Fig. 6a, b). Following vaccination, antibodies were significantly “back-boosted” against these viruses and induced to future virus HK14 (*p* < 0.05). Interestingly, LAIV induced antibodies against the PE09 virus (*p* = 0.027) in the older children born 1995-2002, despite no pre-existing antibodies towards this strain. Adults had higher and broader cross-reactive antibody responses compared to children. There were significant differences between adults and children against viruses that circulated before children were born (HK68-FU02) (Supplementary Table 6). Only the 1967-1976 birth cohort had significantly higher titres than children cohorts post-exposure against all viruses, including the newer strains (CA04-HK14).

Pre-existing titres were similar against recently circulating strains from SY97 to HK14 between the different adult birth cohorts, but differed against historical strains (Fig. 6c–e, Supplementary Table 6). The two older birth cohorts (1948-1966 and 1967-1976) had higher pre-existing titres to HK68 and VI75 than the youngest adults, which circulated before this cohort was born. The 1967-1977 birth cohort had broader antibody responses post-exposure, against 10 of 14 viruses (9 viruses *p* < 0.05 and 1 virus *p* = 0.055), including the oldest HK68 (*p* = 0.046). In contrast, the antibodies in the youngest and oldest adults were boosted primarily against the newer viruses (Fig. 6c–e), back to SY97 or CA04, respectively. The 1967-1976 birth cohort had higher titers post-exposure against HK14, BE92, BE89 and BK79 (*p* < 0.1) compared to the 1948-1966 birth cohort, who did not have the advantage of early childhood H3-priming.

### HI-antibody responses were broadened by age and vaccination

**Fig. 7:**
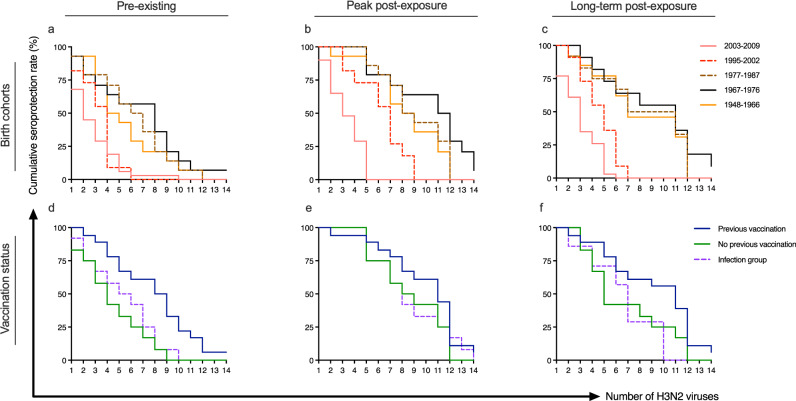
The impact of priming and previous vaccination on the breadth of hemagglutination inhibition (HI) A/H3N2-specific antibody responses. The cumulative seroprotection rates (cumulative percentages of individuals with HI titres ≥40) against a range of influenza A/H3N2 viruses (total 14 viruses) regardless of the viruses’ circulation time pre-exposure (pre-vaccination or infection) (*n* = 84) (**a**), peak responses post-exposure (day 21/28 post-vaccination or 6 months post-infection) (*n* = 84) (**b**), and long-term post-exposure (6 months post-vaccination or 18 months post-infection) (*n* = 79) (**c**). Subjects were stratified by their birth year based on the likelihood of priming with different influenza A subtypes (**a–c**). Children were divided into two birth cohorts: 2003-2009 and 1995-2002 (both H1/H3 primed) and adults were divided into 3 birth cohorts: 1977-1987 (H1/H3 primed), 1967-1976 (H3 primed) and 1948-1966 (H1/H2 primed). The cumulative seroprotection rates against the numbers of influenza A/H3N2 viruses in adults pre-exposure (*n* = 42) (**d**), peak post-exposure (*n* = 42) (**e**), and long-term post-exposure (*n* = 37) (**f**) were stratified by previous vaccination history and compared to the infection group.

To further explore the lifetime impact of influenza A/H3N2-exposure, we calculated the seroprotection rates (percentages of individuals with HI titres ≥40) against the 14 A/H3N2 strains in the five birth cohorts (Supplementary figure 5a–f). We then plotted the cumulative seroprotection rates against the number of viruses pre- and post-exposure (Fig. 7a–c). These seroprotection curves aided in illustrating the breadth of antibody responses against the range of 1-14 A/H3N2-viruses, regardless of when they circulated. We found that the adults had significantly higher seropositivity pre-exposure compared to children, particularly the 1967-1976 birth cohort (Fig. 7a, Supplementary Table 7). The greatest differences pre-exposure between the birth cohorts were observed to 5 viruses, to which ≥43% of adults had protective antibodies, compared to only 6-9% of children. The seroprotection curves were expanded in all birth cohorts after exposure, although the adults had significantly higher percentages of seroprotection than children (Fig. 7b, c, Supplementary Table 7). The 1995-2002 birth cohort had a peak post-exposure seroprotection curve similar to adults up to 7 viruses (Fig. 7b), but did not maintain the seropositivity long-term (Fig. 7c). Importantly, the 1967-1976 birth cohort had broader seroprotection curves after exposure than the 1948-1966 birth cohort (peak *p* = 0.049, long-term p = 0.078), and only this H3-primed cohort had protective antibodies to ≥12 viruses (Fig. 7b, c).

Similarly, we investigated the impact of previous vaccination on the breadth of antibody responses by comparing cumulative seroprotection rates against the range of 1-14 A/H3N2 viruses between adults with different previous vaccination status (Fig. 7d–f) (see Supplementary figure 5g–I for seroprotection rates against each A/H3N2 virus). The children were not included since healthy children are not recommended for annual influenza vaccination in Norway (only 2/42 of children had previously been vaccinated). We found that adults with prior influenza vaccination had a significantly broader seroprotection curve than the previously unvaccinated or infected adults before exposure (Supplementary Table 7, Fig. 7d). The biggest difference pre-exposure was found against 8 viruses, where 50% of the previously vaccinated adults were seropositive compared to only 8% of the previously unvaccinated or infected adults. No significant differences between the groups were found after exposure (Fig. 7e, f). However, we observed a trend of higher percentages of previously vaccinated adults having protective antibodies long-term post-exposure compared to the previously unvaccinated (*p* = 0.068) or infected (*p* = 0.135) adults (Fig. 7f), suggesting a better maintenance of protective antibodies after repeated vaccination.

## Discussion

Since its appearance in 1968, the influenza A/H3N2 has undergone frequent antigenic drift, outpacing vaccine strain selection and reducing seasonal VE. Therefore, studies of antibody landscapes against historical and recent circulating A/H3N2 viruses are important to understand the effect of pre-existing immunity and priming-related differences of antibody responses to current virus exposure. To the best of our knowledge, our study is unique in describing the breadth, magnitude, and durability of antibody responses to 14 historical, recent, and future A/H3N2 viruses after LAIV in children, compared to after IIV or infection in adults. We found that antibody landscapes after vaccination or infection are affected by pre-existing immunity, shaped by priming, prior vaccination status and age. Pre-existing antibodies were found against viruses dating back to the individual’s birth year, in line with earlier studies^16,27^. The pattern of antibody responses post-vaccination or infection followed the pre-existing antibodies. However, the greatest antibody increase post-exposure was observed against viruses that circulated within the last 5-8 years, regardless of priming pattern, vaccination or infection status, or pre-existing immunity. Importantly, antibodies were induced to future strains which the individuals had not yet encountered, potentially providing partial immunity toward future A/H3N2 strains. Remarkably, the exclusively H3-primed adult group had increases in antibodies to most viruses post-exposure, indicating that childhood priming increases the breadth and magnitude of antibody cross-reactivity. Overall, the durability of antibody responses was positively impacted by childhood H3-priming and repeated vaccination, as reflected by broader antibody responses. Our study has implications for the development of next-generation influenza vaccines, and ultimately universal influenza vaccines that can provide antibody-based protection against past and future viruses.

Our findings of narrower antibody responses to A/H3N2 viruses in children agreed with previous findings^31,32^ since age is associated with greater influenza exposure^33^. Vaccination broadened the antibody responses in children, however, adults had maintained broader antibody responses. Importantly, we showed that higher proportions of adults who were previously vaccinated had protective antibodies pre- and post-exposure against higher numbers of A/H3N2 viruses, compared to adults who had not been vaccinated previously or were infected. This suggests that annual influenza vaccination expanded and maintained the breadth of antibody responses as vaccinated individuals had pre-existing protective antibodies and durable post-vaccination antibodies. Unvaccinated individuals with no or low pre-existing antibodies remain more susceptible to influenza infection in the coming seasons as shown in our infection group.

We found that the reactivity to the oldest viruses increased after vaccination or infection, although non-significantly, and did not change the overall antibody landscape. In contrast to the theories of “original antigenic sin” and “antigenic seniority” we found that the more recently circulating viruses (SY97-HK14) dominated the cross-reactive HI-antibody responses in all birth cohorts with higher titres post-exposure. These viruses are more genetically and antigenically similar to the vaccine or infecting viruses than the oldest viruses (Supplementary Figure 1). Our observations best fit a cross-reactive hypothesis, boosting a homologous response, as well as antibodies directed against conserved HA-head epitopes on other strains^26^. Despite the constant evolution of the A/H3N2-viruses, some conserved epitopes remain in the head region, allowing for cross-reactive antibody responses^34^. Whilst we would expect cross-reactivity against TX12 to be induced by the closely related PE09, antibodies were also elicited against the future drifted HK14 virus that dominated from 2015 to 2018. However future studies are needed to confirm cross-reactive antibody responses to circulating viruses^35,36^. On the other hand, the H3-primed adults back-boosted antibodies against most viruses, even the oldest HK68, thus the cross-reactive hypothesis alone does not fully explain our results. Long-term memory responses are probably influenced by multiple mechanisms, including the order of influenza infections^37^. Our findings agree with previous reports of back-boosting^25,27,28^, and further extend the theory by detecting antibodies to an advanced, future strain. More studies of antibody landscapes are needed to better understand the impact of pre-existing immunity on antibody responses. It remains to be seen if our findings extend to different populations and with larger sample sizes.

With occasional antigenic shift and continuous drift, adults and children will have different priming patterns to influenza A subtypes, according to their birth cohorts. The children’s landscapes reflect their infection history, since healthy Norwegian children are rarely recommended for influenza vaccination (only 0.05% in our cohort). The younger children had pre-existing antibodies against the newer strains PE09-HK14, whereas the older children had pre-existing titres to the older strains SY97-WI05, which circulated in their early childhood. This suggests that older children might not be susceptible to influenza infection with the same subtype for a number of years after their first childhood infection^21^. It is unclear how the children’s landscapes will evolve in the future, especially since LAIV-induced immunity is multifaceted and different to antibody-mediated immunity after IIV. We propose that there may be an advantage of childhood A/H3N2 vaccination upon antibody responses in the future. Interestingly, the PE09-unprimed older children clearly demonstrated induction of a cross-reactive antibody response to this strain after LAIV with TX12. This finding supports the vaccine strategy of pre-emptive vaccine updates with an advanced A/H3N2 strain to potentially provide the dual benefit of cross-reactive antibody responses directed to both advanced and previous viruses^25^.

The A/H3N2 viruses have been associated with the lowest VE of all vaccine strains^14,38^, and reduced VE after repeated vaccination^39,40^. However, we found that vaccination-elicited A/H3N2-specific antibody responses were equivalent to infection, suggesting a robust vaccine response. We further observed that repeated seasonal vaccination induced more durable cross-reactive antibody responses than in singly vaccinated individuals or those without previous influenza vaccination, suggesting a potential advantage of repeated vaccination. This discrepancy could be due to the way VE is measured by the test-negative study design, which is largely biased to symptomatic patients requiring medical attention^41,42^. Whereas our findings suggest that repeatedly vaccinated individuals may be partially protected against severe disease and therefore experience asymptomatic or mild infection not seeking medical care. Our healthy adult cohort was <65 years old, a group with higher vaccine performance than the elderly who often experience the greatest burden of A/H3N2 infections. Our results are in line with previous studies, demonstrating that repeated vaccination did not reduce influenza vaccine protection^43–45^ and beneficially induced antibody responses to drifted strains^46^. However, we did not directly evaluate the vaccine protection, but rather measured seroprotection. We suggest that previous vaccination history and priming effects should be accounted for in future VE studies.

Caveats to our study are limited numbers of individuals, although we have included both adults and children, but not the elderly. Furthermore, we used the dominant circulating strains and defined infection by seroconversion at 6-9 months post-infection, since we did not have clinical or laboratory confirmation, limiting the number of subjects in the infection group. We used the HI assay to measure protective antibodies against the head region of HA against 14 A/H3N2 viruses, since only this assay has established correlates of protection. However, IIV-induced antibodies are known to be strain- and HA head-specific, whereas infection and LAIV immunisation elicit a multifaceted response directed against different viral antigens, which are not measured by the HI assay. We cannot exclude all evidence of “original antigenic sin” in our population, as we did not measure HA stalk-targeting antibodies. In addition, detection of traditional HI antibodies is becoming an increasing problem with the most recent A/H3N2 strains^47^, although we found no differences in HI titres against the newest virus (HK14) using an egg-grown virus and different species’ red blood cells (Supplementary Figure 6). Since egg-adapted changes in HK14 vaccine viruses have been reported to alter antigenicity impacting vaccine-induced antibody responses in different age groups^35,36^, our results should be interpreted with caution. Further studies are warranted to investigate the clinical impact of cross-reactive antibody responses upon protection from infection.

Our results show that antibody landscapes diversified with increasing age. However, not only age, but multiple factors, such as the order and variability of influenza exposure influences the antibody back-boosting responses. Individual landscapes displayed great diversity and were not simply guided by the first or successive influenza infections or vaccinations, although priming-related factors were observed in different birth cohorts. More durable HI antibodies were detected after repeated vaccination, and a history of multiple vaccinations broadened the HI antibodies and increased the pool of cross-reactive HI antibody responses. These antibodies might provide partial immunity against novel influenza viruses or the recurring problem of influenza A/H3N2 antigenic drift during an influenza season. Vaccination with A/H3N2 strains in childhood and repeated vaccination with advanced drifted strains may improve vaccine protection against this subtype.

## Methods

### Study design

Adults (21-61 years old) and children (3-17 years old) (*n* = 42 per group) were included in this study which was approved by the regional ethics committee (Regional Committee for Medical Research Ethics, Western Norway (2009/1224 and 2012/1088) and the Norwegian Medicines Agency (National Institute for Health database Clinical trials.gov (NCT01003288 (adults) and NCT01866540 (children)). All participants provided written informed consent. The study population was retrospectively selected to compare A/H3N2-specific antibody responses after IIV, LAIV and infection (Fig. 1a). The majority of adults (32/42) and half of children (20/42) were female (Supplementary Table 1). Depending upon their seasonal vaccination history, adults were divided into three groups (*n* = 10/group): single vaccination in 2010 or 2013, or double vaccination in 2010 and 2013 IIV^30^. Unvaccinated adults who were infected with circulating A/H3N2 viruses were included for comparison (infection group, *n* = 12). Children were intranasally immunized with seasonal LAIV either in 2012 (2012 LAIV, *n* = 22) or in 2013 (2013 LAIV, *n* = 20)^29^.

### Vaccines

The trivalent seasonal IIV was either subunit (Influvac, Abbott Laboratories) or split-virion (Vaxigrip, Sanofi Pasteur) containing 15 μg HA per strain. The trivalent LAIV contained 10^7^ fluorescent focus units (FFU) of each strain (FLUENZ, AstraZeneca). The A/H3N2 viruses changed between seasons from A/Perth/16/2009 in 2010-11 and 2011-12 to A/Victoria/361/2011 in 2012-13 and 2013-14.

### Blood samples

Serum samples were collected pre- and post-vaccination (21 days, 6 and 12 months) in the three IIV adult groups and once a year before the start of influenza season (September/October) in the infected adults. Five adults in the single 2010 IIV group provided long-term follow-up blood samples at 36 and 48 months after vaccination. Plasma samples were collected pre- and post-vaccination (28 and 56 days, 6 and 12 months) in the children (Fig. 1a). Blood samples were aliquoted and stored at −80 °C until used in the HI assay.

### Viruses

Fourteen genetically and antigenically different A/H3N2 viruses were included in the study, spanning from 1968 to 2018 (Fig. 1b, Table 1, Supplementary Figure 1). The wild-type A/Hong Kong/1/1968 (HK68) virus was propagated in embryonated hen eggs and used in the HI assay. The twelve previously circulating viruses and one future strain A/Hong Kong/4801/2014 (HK14) were inactivated egg-grown viruses, derived from either reassortant vaccine strains or reference wild-type viruses (obtained from the National Institute for Biological Standards and Controls (NIBSC), UK or the International Reagent Resource (IRR), USA).

### Hemagglutination inhibition assay

The HI assay was conducted as previously described^30^. Briefly, blood samples were treated with receptor-destroying enzyme (Seiken, Japan) and pre-adsorbed with packed turkey red blood cells (TRBC) before serial dilution from 1/10 in duplicate and incubation with 4 hemagglutinating units of each virus and 0.5% (volume/volume) TRBC. The HI titre was read as the reciprocal of the highest dilution that inhibited 100% hemagglutination. Negative values were assigned a titre of 5 for calculation purposes. GMTs were calculated from duplicates and reported as final titre for each sample. Fourfold or higher increases in HI titres were considered seroconversion.

### Statistical analysis

HI data was analysed and visualized in Prism version-9 (GraphPad Software, USA). Missing data at 6 months were interpolated for subjects that had day-21 and 12-month data using linear regression models (*n* = 6). Other missing data at random were interpolated using the group’s GMT (*n* = 6). Non-parametric ANOVA Friedman, Wilcoxon test or Kruskal-Wallis test were used as appropriate to compare within or between groups, with multiple comparison correction. Log-rank Mantel-Cox test was used to compare the cumulative seroprotection curves between groups. Exact p-values and other statistical values are reported in Supplementary Tables 2-7. *P* < 0.05 was considered statistically significant.

### Reporting Summary

Further information on research design is available in the Nature Research Reporting Summary linked to this article.

## Supplementary information

Supplementary Information

REPORTING SUMMARY

## Data Availability

The datasets generated during and/or analysed during the current study are available from the corresponding authors on reasonable request. Supplementary Figures 2–3 show associated raw data.
