# Supplementary material for: Seasonal influenza vaccination expands hemagglutinin-specific antibody breadth to older and future A/H3N2 viruses
Source: NPJ Vaccines. 2022 Jun 24;7:67. doi: 10.1038/s41541-022-00490-0 (PMC9232600; doi:10.1038/s41541-022-00490-0)
Supplement: Supplementary file 1 — Supplementary Information [file 41541_2022_490_MOESM1_ESM.pdf]

Seasonal influenza vaccination expands heamagglutinin-specific antibody breadth to older and future A/H3N2 viruses

Nina Urke Ertesvåg[1,*], Rebecca Jane Cox[1,3], Sarah Larteley Lartey[1], Kristin Greve-Isdahl Mohn[1,2], Karl Albert Brokstad[1], Mai-Chi Trieu[1,*]

[1]*Influenza Centre, Department of Clinical Science, University of Bergen, Bergen, Norway*
[2]*Department of Medicine, Haukeland University Hospital, Bergen, Norway*
[3]*Department of Microbiology, Haukeland University Hospital, Bergen, Norway*

Supplementary file

Supplementary Tables 1-7

Supplementary Figures 1-6

Supplementary Table 1: Demographics of the study population

Characteristics	Adults					Children		
	**Total** (n=42)	**Infection** (n=12)	**2010 IIV** (n=10)	**2013 IIV** (n=10)	**2010 & 2013 IIV** (n=10)	**Total** (n=42)	**2012 LAIV** (n=20)	**2013 LAIV** (n=22)
Age (median ± SD)	36.0 ± 9.6	33.5 ± 8.3	34.5 ± 7.7	46.0 ± 11.1	37 ± 9.1	5.0 ± 5.2	5.0 ± 5.5	5.0 ± 4.9
Sex (Female/Male)	32/10	9/3	7/3	9/1	7/3	20/22	8/12	12/10
High-risk condition*	1 (2.4%)	0 (0%)	0 (0%)	0 (0%)	1 (10%)	-	-	-
Previous seasonal vaccination	22 (52.4%)	3 (25%)	6 (60%)	8 (80%)	5 (50%)	-	-	-

*Pregnancy, chronic respiratory diseases, neurological diseases, immunosuppressive diseases, heart diseases and obesity.
- Not applicable for children.
A total of 42 adults and 42 children were included in the study.

Supplementary Table 2: Statistical analysis for Figure 2

Analysis		Multiple comparison		Friedman test	
		Z value	Adjusted p value	Friedman statistics	P value
**PE09 infected (n=7), Fig 2a**					
	Pre inf vs 6M	4.244	**<0.0001**	19.46	**0.0002**
	Pre inf vs 18M	2.484	**0.0389**		
**TX12 infected (n=5), Fig 2b**					
	Pre inf vs 6M	-	0.0625*	-	-
**Single IIV 2010 (n=10), Fig 2c**					
	D0 vs D21	4.850	**<0.0001**	27.60	**<0.0001**
	D0 vs 6M	3.291	**0.0030**		
**Single IIV 2013 (n=10), Fig 2d**					
	D0 vs D21	3.811	**0.0004**	20.01	**0.0002**
**Double IIV 2010 (n=10), Fig 2e**					
	D0 vs D21	3.897	**0.0003**	20.09	**0.0002**
	D0 vs 6M	2.425	**0.0459**		
**Double IIV 2013 (n=10), Fig 2f**					
	D0 vs D21	3.724	**0.0006**	22.07	**<0.0001**
**LAIV 2012 (n=22), Fig 2g**					
	D0 vs D28	5.438	**<0.0001**	50.35	**<0.0001**
	D0 vs D56	4.258	**<0.0001**		
	D0 vs 6M	2.616	**0.0356**		
**LAIV 2013 (n=20), Fig 2h**					
	D0 vs D28	3.855	**0.0005**	31.67	**<0.0001**
	D0 vs D56	3.025	**0.0099**		

*Two-tailed exact p value from Wilcoxon test.

Data was analysed using a matched, repeated measure, non-parametric Friedman's test with Dunn's multiple comparison test, except the TX12 infected group due to missing samples for long-term follow-up. The TX12 infected group was analysed using non-parametric Wilcoxon matched-pairs signed rank test for pre-infection and 6-9M post infection time points.

Supplementary Table 3: Statistical analysis for Figure 3 and 4

Analysis			Multiple comparison		Friedman test	
			Z value	Adjusted p value	Friedman statistics	P value
**PE09 infected**						
	**Fig 3a (n=7)**					
	**Pre inf vs 6M**					
		HK14	3.519	**0.0013**	17.21	**0.0006**
		TX12	4.347	**<0.0001**	20.45	**0.0001**
		PE09	4.244	**<0.0001**	19.46	**0.0002**
		WI05	3.726	**0.0006**	18.78	**0.0003**
	**Fig 3b (n=7)**					
	**Pre inf vs 18M**					
		TX12	2.484	**0.0389**	20.45	**0.0001**
		PE09	2.484	**0.0389**	19.46	**0.0002**
		WI05	2.588	**0.0290**	18.78	**0.0003**
**TX12 infected**						
	**Fig 3d (n=5)**					
	**Pre inf vs 6M**					
		TX12	-	0.0625*	-	-
**2010 IIV (n=10), Fig 4a**						
	**D0 vs D21**					
		HK14	3.897	**0.0003**	21.48	**<0.0001**
		TX12	4.850	**<0.0001**	28.16	**<0.0001**
		PE09	4.850	**<0.0001**	27.60	**<0.0001**
		WI05	4.590	**<0.0001**	25.84	**<0.0001**
		CA04	3.811	**0.0004**	18.80	**0.0003**
		FU02	3.984	**0.0002**	20.84	**0.0001**
		SY97	2.858	**0.0128**	16.14	**0.0011**
	**D0 vs 6M**					
		HK14	2.511	**0.0361**	21.48	**<0.0001**
		TX12	3.551	**0.0012**	28.16	**<0.0001**
		PE09	3.291	**0.0030**	27.60	**<0.0001**
		WI05	4.590	**0.0361**	25.84	**<0.0001**
**2013 IIV (n=10), Fig 4b**						
	**D0 vs D21**					
		HK14	3.464	**0.0016**	19.64	**0.0002**
		TX12	3.811	**0.0004**	20.01	**0.0002**
		PE09	3.811	**0.0004**	20.93	**0.0001**
		WI05	3.118	**0.0055**	15.64	**0.0013**
		CA04	2.858	**0.0128**	13.88	**0.0031**
**Double 2010 IIV (n=10), Fig 4c**						
	**D0 vs D21**					
		HK14	3.204	**0.0041**	16.54	**0.0009**
		TX12	4.070	**0.0001**	21.19	**<0.0001**
		PE09	3.897	**0.0003**	20.09	**0.0002**
		WI05	3.724	**0.0006**	17.23	**0.0006**
		CA04	2.944	**0.0097**	14.10	**0.0028**
	**D0 vs 6M**					
		PE09	3.897	**0.0459**	20.09	**0.0002**
**Double 2013 IIV (n=10), Fig 4d**						
	**D0 vs D21**					
		TX12	3.724	**0.0006**	22.07	**<0.0001**
		PE09	3.377	**0.0022**	18.63	**0.0003**
		WI05	2.944	**0.0097**	14.82	**0.0020**
		CA04	2.858	**0.0128**	13.70	**0.0034**

*Two-tailed exact p value from Wilcoxon test.

Data was analysed using a matched, repeated measure, non-parametric Friedman's test with Dunn's multiple comparison test, except the TX12 infected group due to missing samples. The TX12 infected group was analysed using non-parametric Wilcoxon matched-pairs signed rank test for pre infection and 6-9M post infection time points.

Supplementary Table 4: Statistical analysis for Figure 5

Analysis			Multiple comparison		Friedman test	
			Z value	Adjusted p value	Friedman statistics	P value
**2012 LAIV (n=20), Fig 5a**						
	**D0 vs D28**					
		HK14	4.207	**0.0001**	26.69	**<0.0001**
		TX12	5.438	**<0.0001**	50.39	**<0.0001**
		PE09	4.720	**<0.0001**	40.63	**<0.0001**
		WI05	3.796	**0.0006**	34.72	**<0.0001**
	**D0 vs D56**					
		HK14	4.300	**<0.0001**	26.69	**<0.0001**
		TX12	4.258	**<0.0001**	50.39	**<0.0001**
		PE09	4.001	**0.0003**	40.63	**<0.0001**
		WI05	4.617	**<0.0001**	34.72	**<0.0001**
		CA04	2.770	**0.0224**	24.10	**<0.0001**
	**D0 vs 6M**					
		HK14	3.642	**0.0011**	26.69	**<0.0001**
		TX12	2.565	**0.0413**	50.39	**<0.0001**
		PE09	2.565	**0.0413**	40.63	**<0.0001**
		WI05	2.924	**0.0138**	34.72	**<0.0001**
	**D0 vs 12M**					
		HK14	2.514	**0.0478**	26.69	**<0.0001**
**2013 LAIV (n=22), Fig 5b**						
	**D0 vs D21**					
		HK14	3.220	**0.0051**	16.30	**0.0026**
		TX12	3.318	**0.0036**	20.61	**0.0004**
	**D0 vs D56**					
		TX12	2.830	**0.0186**	20.61	**0.0004**
	**D0 vs 6M**					
		HK14	2.879	**0.0160**	16.30	**0.0026**

Data was analysed using a matched, repeated measure, non-parametric Friedman's test with Dunn's multiple comparison test.

Supplementary Table 5: Statistical analysis for Figure 6

Analysis		Wilcoxon tests		Summary
		P value	Adjusted p value	
**Born 2003-2009 (n=31), Fig 6a**				
	HK14	0.0014	**0.0042**	**
	TX12	0.0001	**0.0003**	**
	PE09	0.0233	**0.0310**	*
	WI05	0.0001	**0.0002**	**
	CA04	0.0156	**0.0310**	*
**Born 1995-2002 (n=11), Fig 6b**				
	HK14	0.0039	**0.0270**	*
	TX12	0.0020	**0.0155**	*
	PE09	0.0039	**0.0270**	*
	WI05	0.0078	**0.0270**	*
	CA04	0.0039	**0.0270**	*
	FU02	0.0156	**0.0310**	*
	SY97	0.0039	**0.0270**	*
**Born 1977-1987 (n=14), Fig 6c**				
	HK14	0.0005	**0.0024**	**
	TX12	0.0001	**0.0010**	***
	PE09	0.0001	**0.0010**	***
	WI05	0.0001	**0.0010**	***
	CA04	0.0010	**0.0029**	**
	FU02	0.0005	**0.0024**	**
	SY97	0.0137	**0.0272**	*
**Born 1967-1976 (n=14), Fig 6d**				
	HK14	0.0002	**0.0022**	**
	TX12	0.0001	**0.0012**	**
	PE09	0.0002	**0.0022**	**
	WI05	0.0010	**0.0068**	**
	CA04	0.0020	**0.0117**	*
	FU02	0.0020	**0.0117**	*
	SY97	0.0020	**0.0117**	*
	BE92	0.0156	**0.0461**	*
	SI87	0.0547	0.0547	#
	HK68	0.0234	**0.0463**	*
**Born 1948-1966 (n=14), Fig 6e**				
	HK14	0.0040	**0.0078**	**
	TX12	0.0005	**0.0029**	**
	PE09	0.0010	**0.0049**	**
	WI05	0.0020	**0.0078**	**
	CA04	0.0020	**0.0078**	**
	SY97	0.0840	0.0840	#

Statistical analyses were performed with non-parametric Wilcoxon matched-pairs signed rank test with individual ranks computed for each comparison, and Holm-Šídák method for multiple comparisons.

Supplementary Table 6: Statistical analysis of group comparisons for Figure 6

Analysis	Pre-exposure		Post-exposure	
	t value	Adjusted p value	t value	Adjusted p value
**HK14**				
1967-1976 vs. 1948-1966	0.271	ns	2.582	0.0718
1967-1976 vs. 1995-2002	0.642	ns	3.089	**0.0184**
1967-1976 vs. 2003-2009	2.027	ns	3.564	**0.0038**
1995-2002 vs. 2003-2009	2.596	0.0914	0.276	ns
**TX12**				
1967-1976 vs. 1995-2002	0.243	ns	2.753	0.0528
1967-1976 vs. 2003-2009	2.048	ns	2.921	**0.0351**
**PE09**				
1967-1976 vs. 1995-2002	1.277	ns	3.547	**0.0041**
1977-1987 vs. 1995-2002	0.705	ns	3.483	**0.0046**
1967-1976 vs. 2003-2009	1.393	ns	3.325	**0.0073**
1977-1987 vs. 2003-2009	2.109	ns	3.245	**0.0084**
1995-2002 vs. 2003-2009	2.745	0.0599	1.022	ns
**WI05**				
1948-1966 vs. 2003-2009	1.155	ns	3.366	**0.0055**
1967-1976 vs. 2003-2009	1.791	ns	4.163	**0.0003**
1977-1987 vs. 2003-2009	0.757	ns	4.084	**0.0004**
1995-2002 vs. 2003-2009	2.652	0.0783	3.594	**0.0027**
**CA04**				
1948-1966 vs. 2003-2009	2.317	ns	4.482	**<0.0001**
1967-1976 vs. 2003-2009	1.919	ns	4.961	**<0.0001**
1977-1987 vs. 2003-2009	1.442	ns	4.881	**<0.0001**
1995-2002 vs. 2003-2009	2.080	ns	3.873	**0.0008**
**FU02**				
1948-1966 vs. 1995-2002	6.115	**<0.0001**	5.430	**<0.0001**
1967-1976 vs. 1995-2002	5.479	**<0.0001**	5.239	**<0.0001**
1977-1987 vs. 1995-2002	4.907	**<0.0001**	5.175	**<0.0001**
1948-1966 vs. 2003-2009	9.643	**<0.0001**	12.54	**<0.0001**
1967-1976 vs. 2003-2009	8.848	**<0.0001**	12.30	**<0.0001**
1977-1987 vs. 2003-2009	8.132	**<0.0001**	12.22	**<0.0001**
1995-2002 vs. 2003-2009	1.828	ns	5.270	**<0.0001**
**SY97**				
1948-1966 vs. 2003-2009	6.489	**<0.0001**	7.912	**<0.0001**
1967-1976 vs. 2003-2009	6.967	**<0.0001**	8.776	**<0.0001**
1977-1987 vs. 2003-2009	6.887	**<0.0001**	8.870	**<0.0001**
1995-2002 vs. 2003-2009	5.271	**<0.0001**	7.320	**<0.0001**
**WU95**				
1977-1987 vs. 1967-1976	2.982	**0.0203**	2.446	ns
1948-1966 vs. 1995-2002	2.820	**0.0289**	1.820	ns
1977-1987 vs. 1995-2002	4.410	**0.0001**	4.115	**0.0004**
1948-1966 vs. 2003-2009	3.669	**0.0020**	2.975	**0.0237**
1967-1976 vs. 2003-2009	2.158	ns	2.975	**0.0237**
1977-1987 vs. 2003-2009	5.658	**<0.0001**	5.846	**<0.0001**
**BE92**				
1967-1976 vs. 1948-1966	1.559	ns	2.379	0.0517
1977-1987 vs. 1948-1966	2.982	**0.0146**	2.582	**0.0453**
1948-1966 vs. 1995-2002	2.884	**0.0159**	1.936	ns
1967-1976 vs. 1995-2002	4.346	**0.0001**	4.167	**0.0002**
1977-1987 vs. 1995-2002	5.681	**<0.0001**	4.358	**0.0001**
1948-1966 vs. 2003-2009	4.000	**0.0004**	5.265	**<0.0001**
1967-1976 vs. 2003-2009	5.830	**<0.0001**	8.057	**<0.0001**
1977-1987 vs. 2003-2009	7.500	**<0.0001**	8.296	**<0.0001**

	1995-2002 vs. 2003-2009	0.360	ns	2.608	**0.0453**
**BE89**					
	1967-1976 vs. 1948-1966	2.169	0.0882	2.311	0.0618
	1977-1987 vs. 1948-1966	3.117	**0.0093**	3.126	**0.0091**
	1948-1966 vs. 1995-2002	2.763	**0.0231**	2.782	**0.0218**
	1967-1976 vs. 1995-2002	4.797	**<0.0001**	4.949	**<0.0001**
	1977-1987 vs. 1995-2002	5.687	**<0.0001**	5.714	**<0.0001**
	1948-1966 vs. 2003-2009	3.480	**0.0031**	3.988	**0.0004**
	1967-1976 vs. 2003-2009	6.025	**<0.0001**	6.700	**<0.0001**
	1977-1987 vs. 2003-2009	7.139	**<0.0001**	7.658	**<0.0001**
	1995-2002 vs. 2003-2009	0.021	ns	0.466	0.6577
**SI87**					
	1948-1966 vs. 1995-2002	3.560	**0.0019**	3.790	**0.0008**
	1967-1976 vs. 1995-2002	4.450	**<0.0001**	5.448	**<0.0001**
	1977-1987 vs. 1995-2002	5.404	**<0.0001**	5.766	**<0.0001**
	1948-1966 vs. 2003-2009	3.880	**0.0007**	4.729	**<0.0001**
	1967-1976 vs. 2003-2009	4.993	**<0.0001**	6.803	**<0.0001**
	1977-1987 vs. 2003-2009	6.187	**<0.0001**	7.202	**<0.0001**
**BK79**					
	1967-1976 vs. 1948-1966	2.779	**0.0329**	2.445	0.0573
	1948-1966 vs. 1995-2002	1.971	ns	2.898	**0.0190**
	1967-1976 vs. 1995-2002	4.577	**<0.0001**	5.191	**<0.0001**
	1977-1987 vs. 1995-2002	2.861	**0.0298**	3.280	**0.0064**
	1948-1966 vs. 2003-2009	1.963	ns	3.685	**0.0017**
	1967-1976 vs. 2003-2009	5.224	**<0.0001**	6.555	**<0.0001**
	1977-1987 vs. 2003-2009	3.077	**0.0170**	4.163	**0.0003**
**VI75**					
	1967-1976 vs. 1977-1987	3.185	**0.0118**	3.330	**0.0072**
	1967-1976 vs. 1995-2002	3.514	**0.0041**	3.900	**0.0009**
	1948-1966 vs. 2003-2009	1.642	ns	2.892	**0.0270**
	1967-1976 vs. 2003-2009	4.188	**0.0003**	5.365	**<0.0001**
**HK68**					
	1948-1966 vs. 1967-1976	2.507	0.0600	2.650	**0.0322**
	1948-1966 vs. 1977-1987	4.947	**<0.0001**	5.844	**<0.0001**
	1967-1976 vs. 1977-1987	2.440	0.0600	3.194	**0.0072**
	1948-1966 vs. 1995-2002	5.531	**<0.0001**	6.213	**<0.0001**
	1967-1976 vs. 1995-2002	3.179	**0.0091**	3.726	**0.0012**
	1948-1966 vs. 2003-2009	5.531	**<0.0001**	7.905	**<0.0001**
	1967-1976 vs. 2003-2009	3.905	**0.0007**	4.794	**<0.0001**

Data was analysed using two-way ANOVA and Holm-Sidak's multiple comparisons test with individual variances computed for each comparison.

ns – not significant p value > 0.05

Supplementary Table 7: Statistical analysis of seroprotection for Figure 7

Analysis		Chi-square	P value	Summary
**Figure 7a – Pre-exposure**				
	1967-1976 vs. 1995-2002	7.189	**0.0073**	**
	1948-1966 vs. 2003-2009	8.378	**0.0038**	**
	1967-1976 vs. 2003-2009	11.95	**0.0005**	***
	1977-1987 vs. 2003-2009	10.45	**0.0012**	**
**Figure 7b – Peak post-exposure**				
	1967-1976 vs. 1948-1966	3.861	**0.0494**	*
	1948-1966 vs. 1995-2002	4.616	**0.0317**	*
	1967-1976 vs. 1995-2002	9.858	**0.0017**	**
	1977-1987 vs. 1995-2002	6.660	**0.0099**	**
	1948-1966 vs. 2003-2009	25.28	**<0.0001**	***
	1967-1976 vs. 2003-2009	28.45	**<0.0001**	***
	1977-1987 vs. 2003-2009	30.21	**<0.0001**	***
	1995-2002 vs. 2003-2009	16.05	**<0.0001**	***
**Figure 7c – Long-term post-exposure**				
	1967-1976 vs. 1948-1966	3.102	0.0782	ns
	1967-1976 vs. 1995-2002	8.747	**0.0031**	**
	1948-1966 vs. 1995-2002	3.956	**0.0047**	*
	1977-1987 vs. 1995-2002	7.727	**0.0054**	**
	1967-1976 vs. 2003-2009	19.59	**<0.0001**	***
	1948-1966 vs. 2003-2009	15.21	**<0.0001**	***
	1977-1987 vs. 2003-2009	20.61	**<0.0001**	***
	1995-2002 vs. 2003-2009	6.972	**0.0083**	**
**Figure 7d – Pre-exposure**				
	Prev vacc vs no prev vacc	8.904	**0.0028**	**
	Prev vacc vs infected	5.526	**0.0187**	*
**Figure 7f – Long-term post-exposure**				
	Prev vacc vs no prev vacc	3.339	0.0677	ns
	Prev vacc vs infected	2.234	0.1350	ns

Data was analysed using log-rank Mantel-Cox test.
ns – not significant p value > 0.05

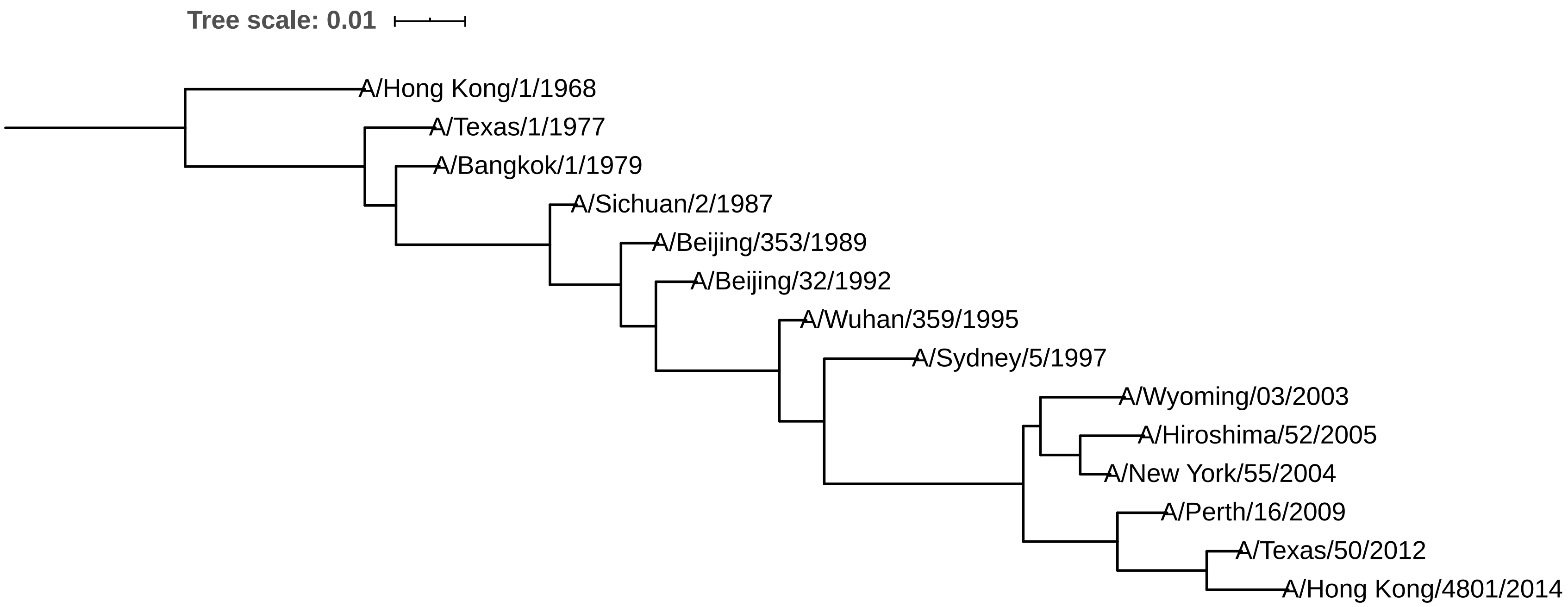

Supplementary Figure 1: Phylogenetic tree of influenza A/H3N2 viruses based on the hemagglutinin (HA) sequence. The HA sequences of 14 influenza A/H3N2 strains circulated from 1968 up until 2018 were obtained from National Center for Biotechnology Information (NCBI, USA, https://www.ncbi.nlm.nih.gov) and Global initiative on sharing all influenza data (GISAID, https://www.gisaid.org). The phylogenetic tree was generated using NGphylogeny.fr, a free online tool from NCBI. HA distances of 0.01 is indicated in the upper left corner.

Supplementary Figure 2: Adult individual landscapes

a) Infection group

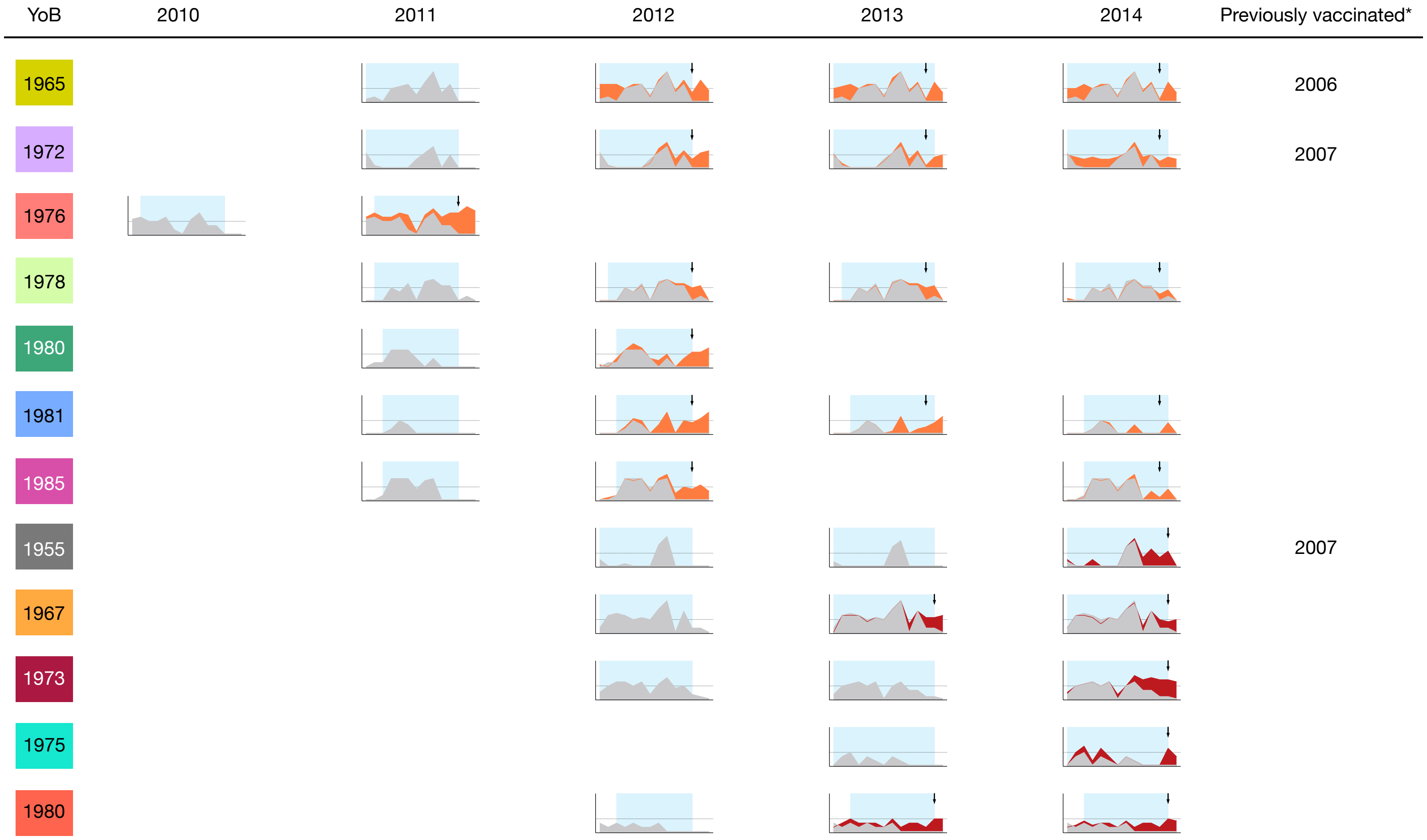

*Last previous seasonal vaccination.

Geometric mean (GM) haemagglutination inhibition (HI) titers are shown on the Y-axis and the 14 antigenically distinct influenza A/H3N2 viruses from old (1968) to new (2018) along the X-axis. The grey shaded area represents pre-infection titers, orange and red represents post-infection titres at up to three different years. Adults were divided into two groups based on the infecting virus: the first 7 individuals were infected with a PE09-like virus in either 2011 or 2012 (orange post-infection titres), the remaining 5 adults were infected with TX12-like virus in 2013 or 2014 (red post-infection titres). The black arrow indicates the infecting virus, and the dotted line indicates the HI titre of 40. The period of viral exposure is highlighted by a light blue background. Adults were arranged according to birth year (left hand side) within each group.

b) Single 2010 IIV

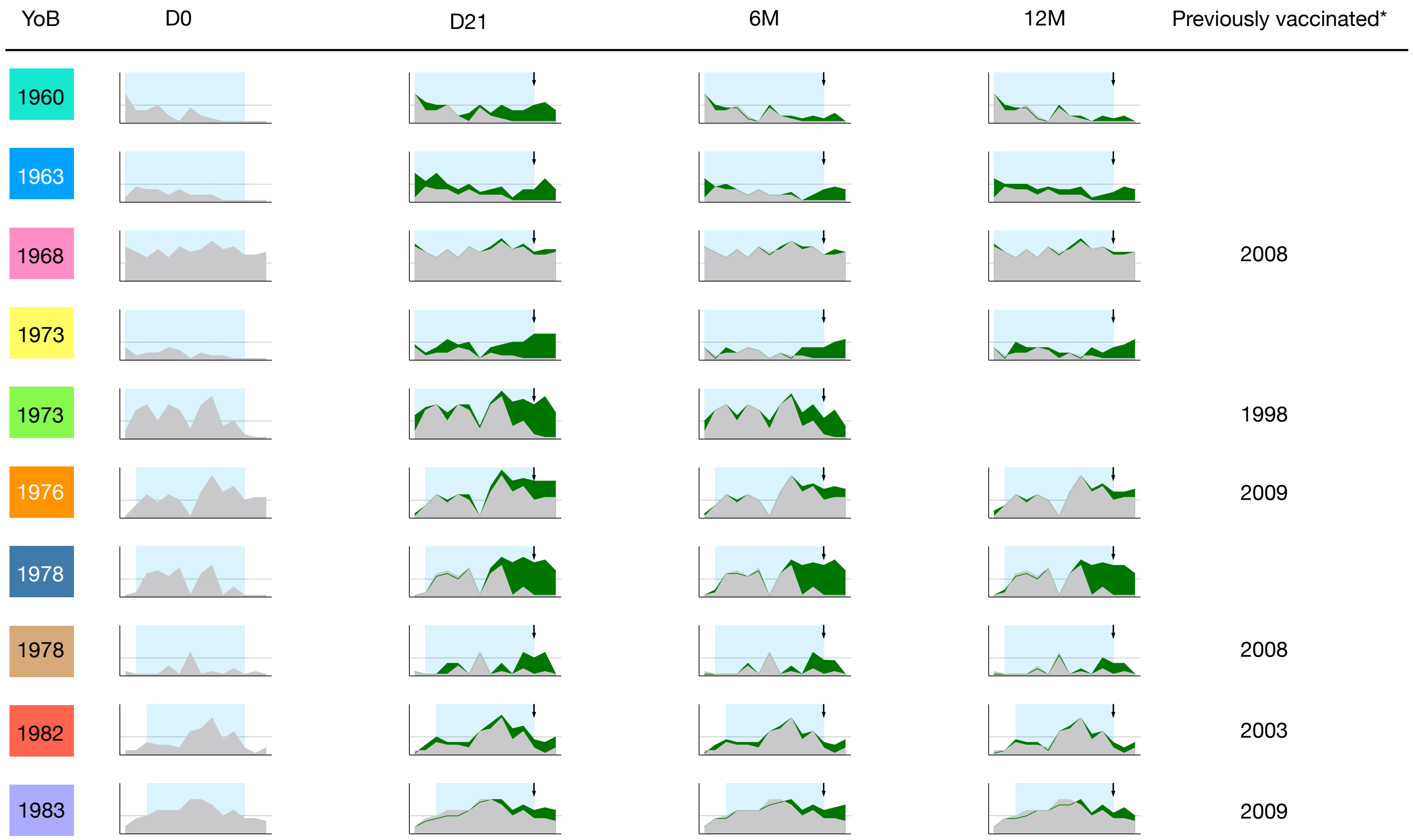

*Last previous seasonal vaccination.

Geometric mean (GM) haemagglutination inhibition (HI) titers are shown on the Y-axis and the 14 antigenically distinct Influenza A/H3N2 viruses from old (1968) to new (2018) along the X-axis. The grey area represents pre-IIV titers at Day 0 (D0), dark green represents post-IIV titres at 21 days (21D), 6 months (6M) and 12 months (12M). Ten adults were included in this group, arranged according to their birth year (left hand side). The black arrow indicates the vaccination virus, and the dotted line indicates the HI titre of 40. The period of viral exposure is highlighted by a light blue background.

c) Single 2013 IIV

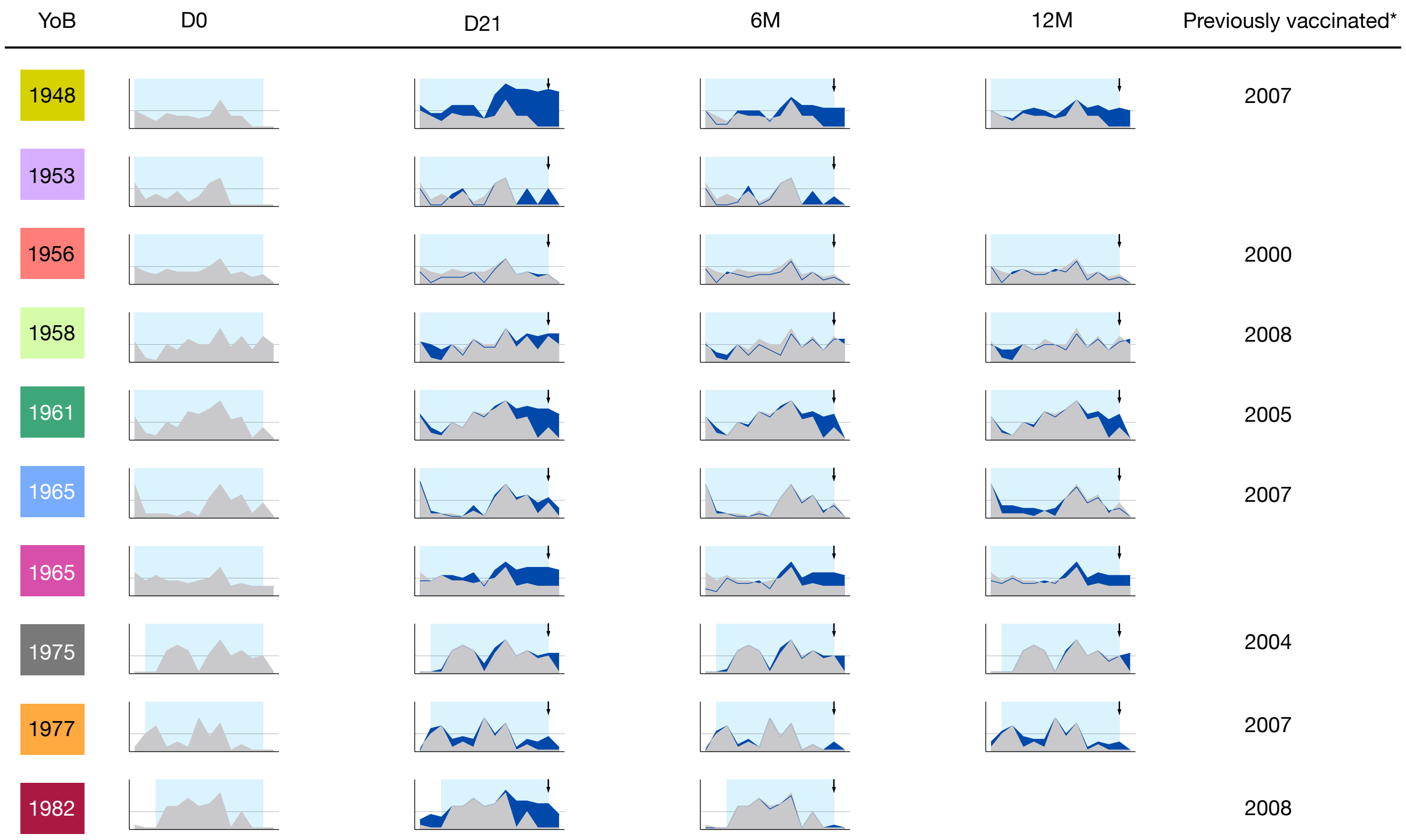

*Last previous seasonal vaccination.

Geometric mean (GM) haemagglutination inhibition (HI) titers are shown on the Y-axis and the 14 antigenically distinct Influenza A/H3N2 viruses from old (1968) to new (2018) along the X-axis. The grey area represents pre-IIV titers, dark blue represents post-IIV titres at 21 days (21D), 6 months (6M) and 12 months (12M). Ten adults were included in this group, arranged according to their birth year (left hand side). The black arrow indicates the vaccination virus, and the dotted line indicates the HI titre of 40. The period of viral exposure is highlighted by a light blue background.

d) Double 2010 & 2013 IIV

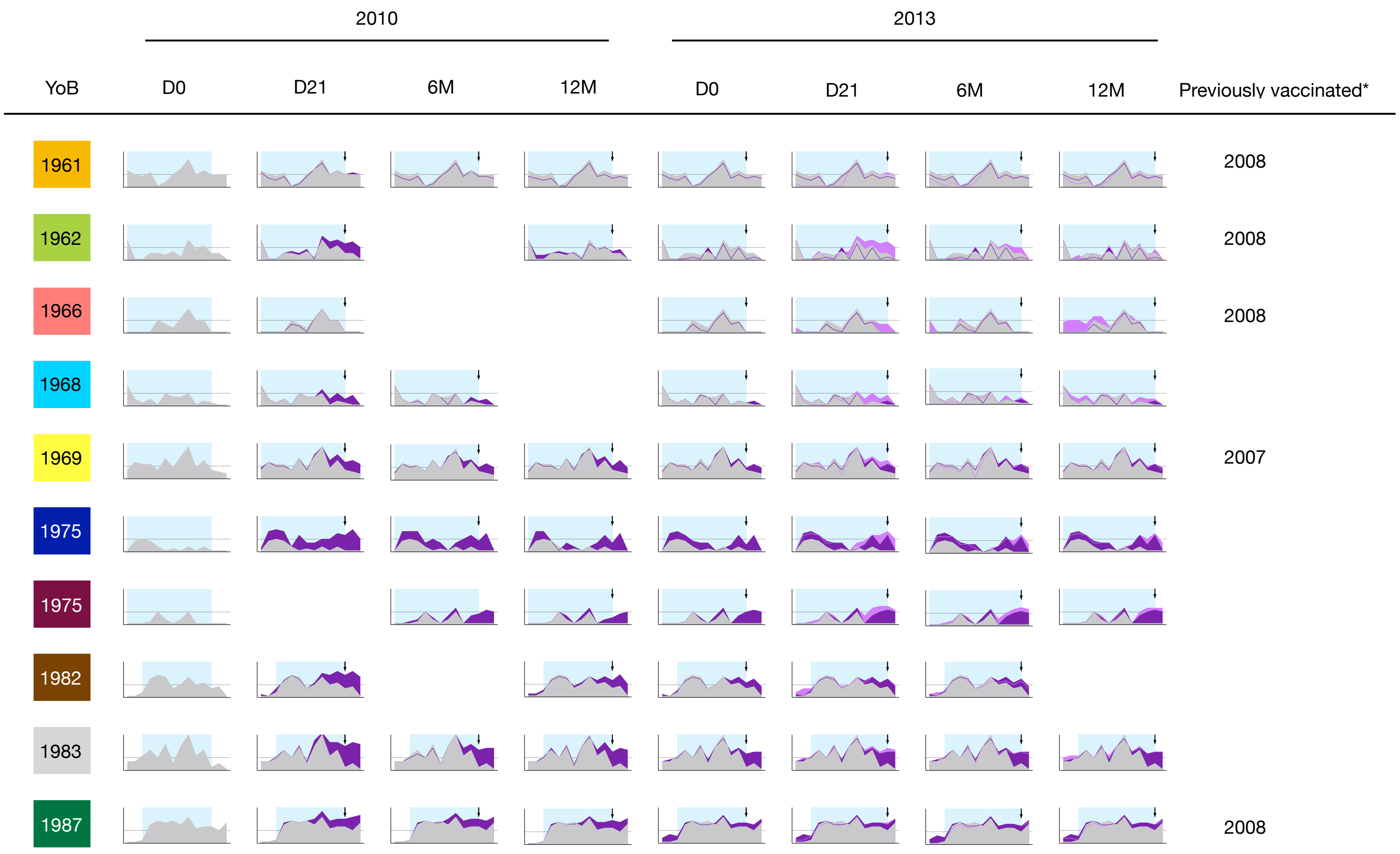

*Last previous seasonal vaccination.

Geometric mean (GM) haemagglutination inhibition (HI) titers are shown on the Y-axis and the 14 antigenically distinct Influenza A/H3N2 viruses from old (1968) to new (2018) along the X-axis. The grey area represents pre-IIV titers at Day 0 (D0) 2010, post-IIV titres in dark purple after 1st vaccination (PE09) 21 days (21D), 6 months (6M) and 12 months (12M).and in light purple after 2nd vaccination (TX12) at D21, 6 and 12M. Ten adults were included in this group, arranged according to birth year (left hand side). The black arrow indicates the vaccination virus, and the dotted line indicates the HI titre of 40. The period of viral exposure is highlighted by a light blue background.

Supplementary Figure 3: Individual landscapes after live attenuated influenza vaccine (LAIV) in children

a) LAIV 2012

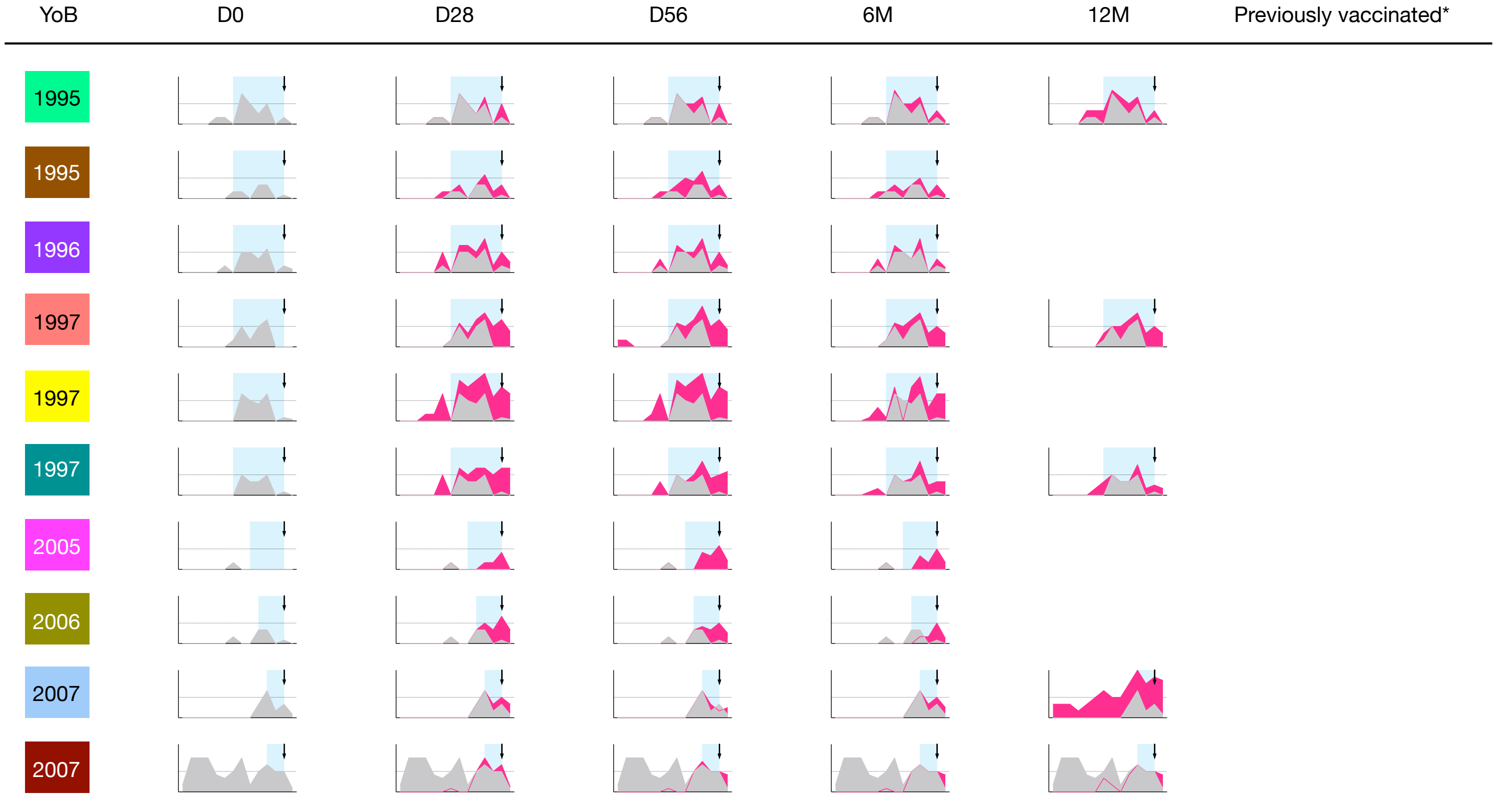

*Previous seasonal vaccination.

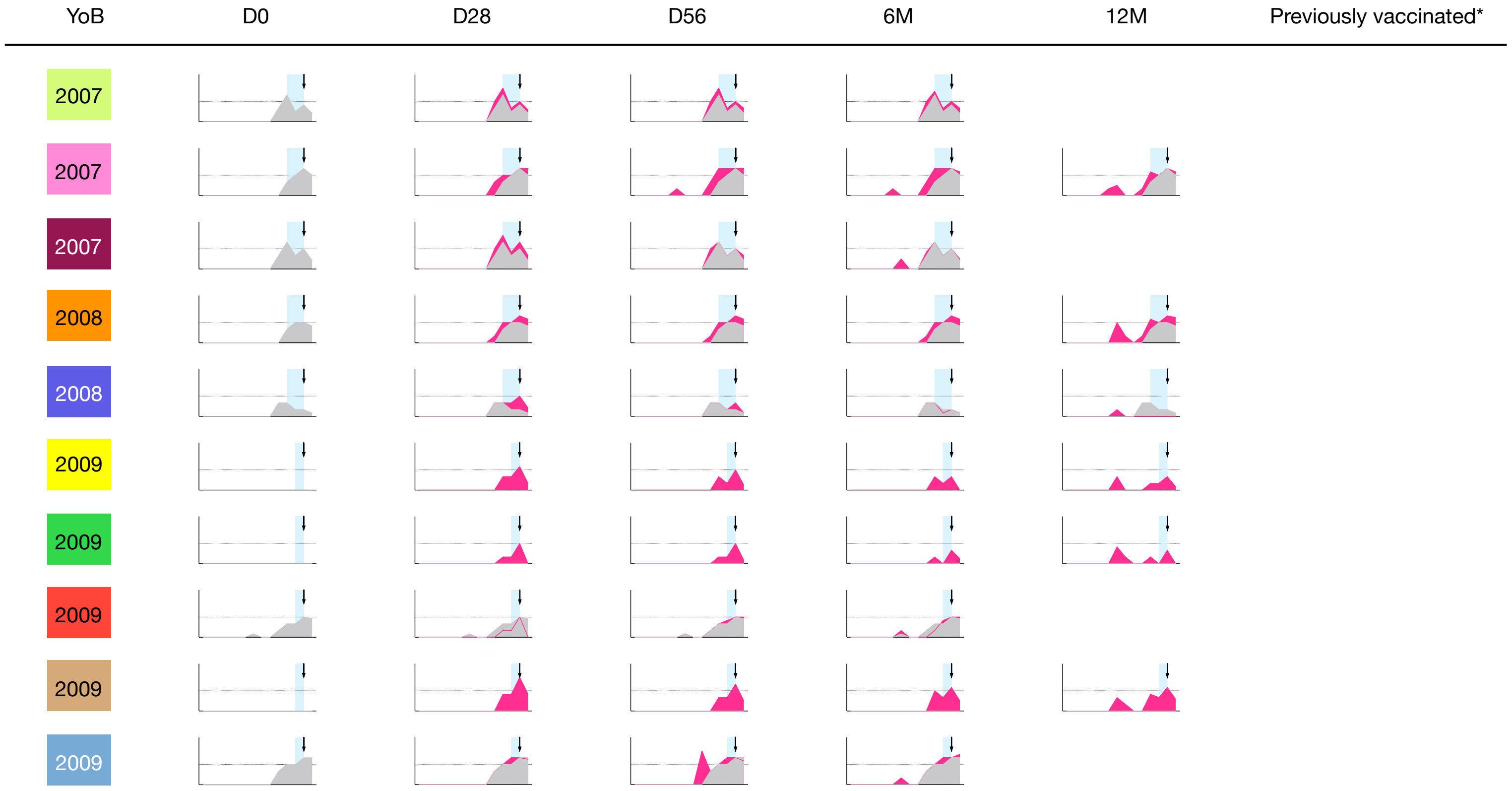

*Previous seasonal vaccination.

Geometric mean (GM) haemagglutination inhibition (HI) titers are shown on the Y-axis and the 14 antigenically distinct Influenza A/H3N2 viruses from old (1968) to new (2018) along the X-axis. The grey area represents pre-LAIV titers (Day 0, D0), pink represents post-IIV titres at 28 days (28D) and 56D, 6 months (6M) and 12 months (12M). Twenty children were included in this group (**a**) 10 and (**b**) 10, arranged according to birth year (left hand side). The black arrow indicates the vaccination virus, and the dotted line indicates the HI titre of 40. The period of viral exposure is highlighted by a light blue background.

b) LAIV 2012

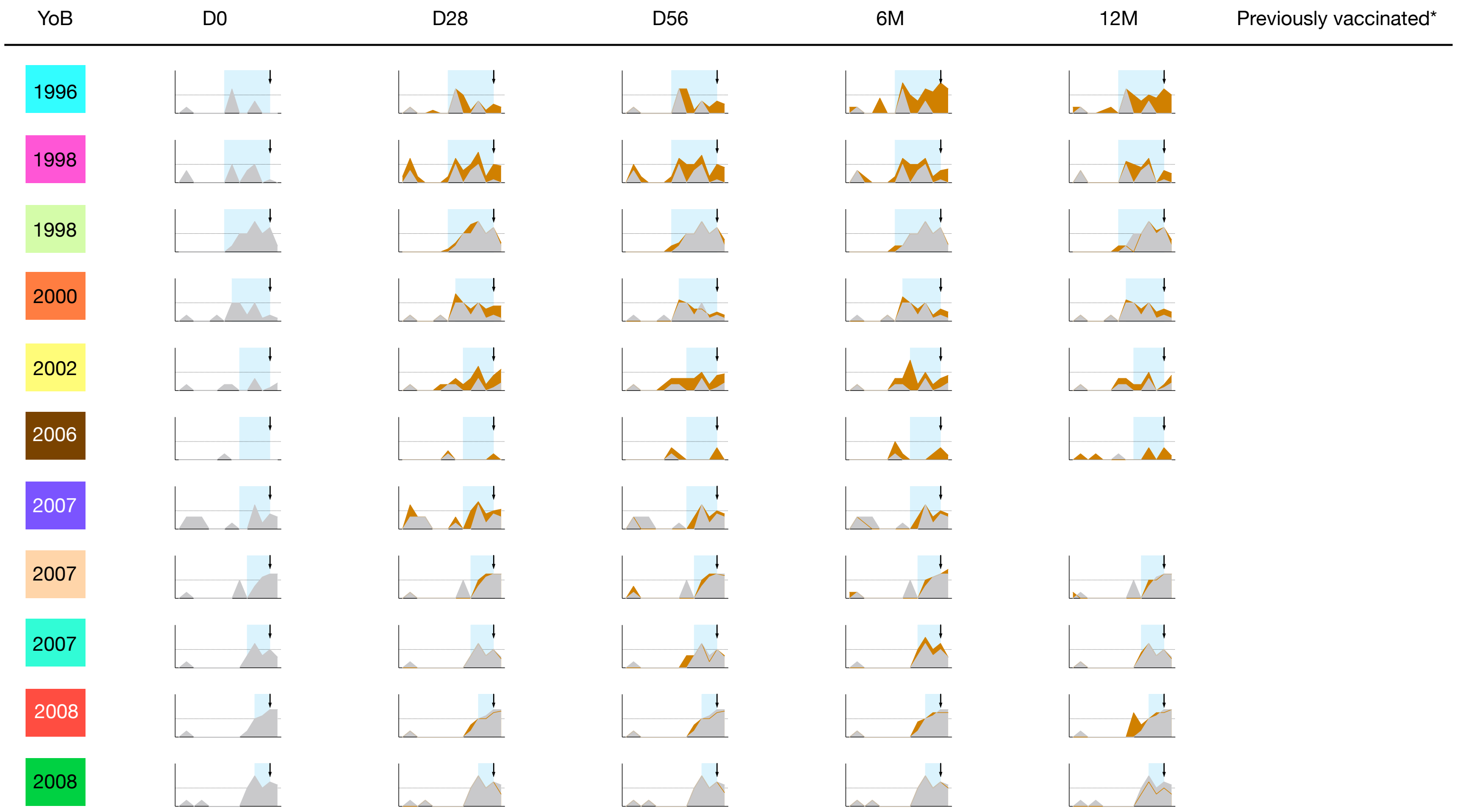

*Previous seasonal vaccination.

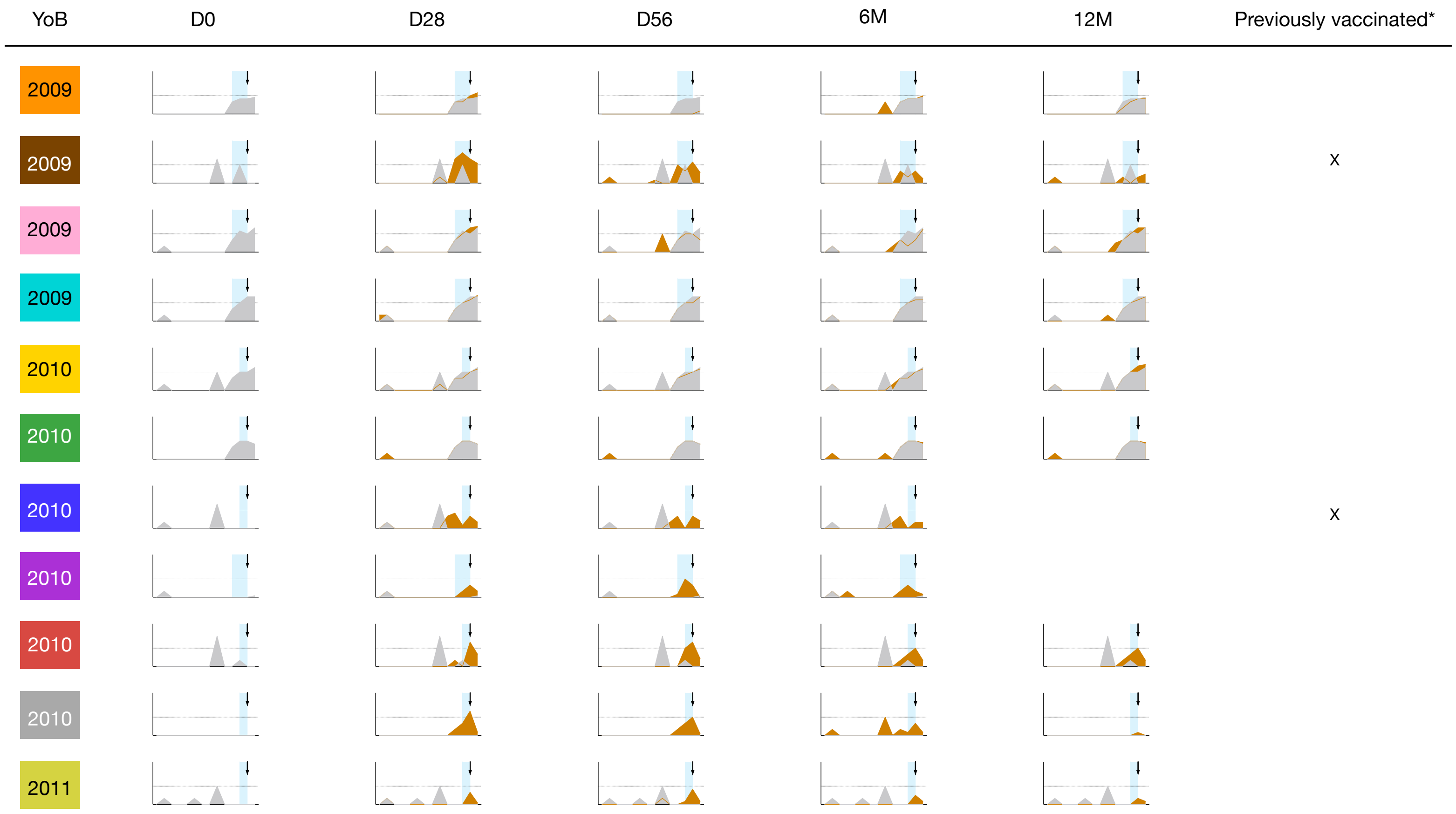

*Previous seasonal vaccination.

Geometric mean (GM) hemagglutination inhibition (HI) titers are shown on the Y-axis and the 14 antigenically distinct Influenza A/H3N2 viruses from old (1968) to new (2018) along the X-axis. The grey area represents pre-LAIV titers Day 0 (D0), pink represents post-IIV titres at 28 days (28D) and 56D, 6 months (6M) and 12 months (12M). Twenty-two children were included in this group (**a**) 11 and (**b**) 11, arranged according to birth year (left hand side). The black arrow indicates the vaccination virus, and the dotted line indicates the HI titre of 40. The period of viral exposure is highlighted by a light blue background.

Supplementary Figure 4: Landscapes of vaccination long-term responses after inactivated influenza vaccine in 2010 (IIV 2010 group)

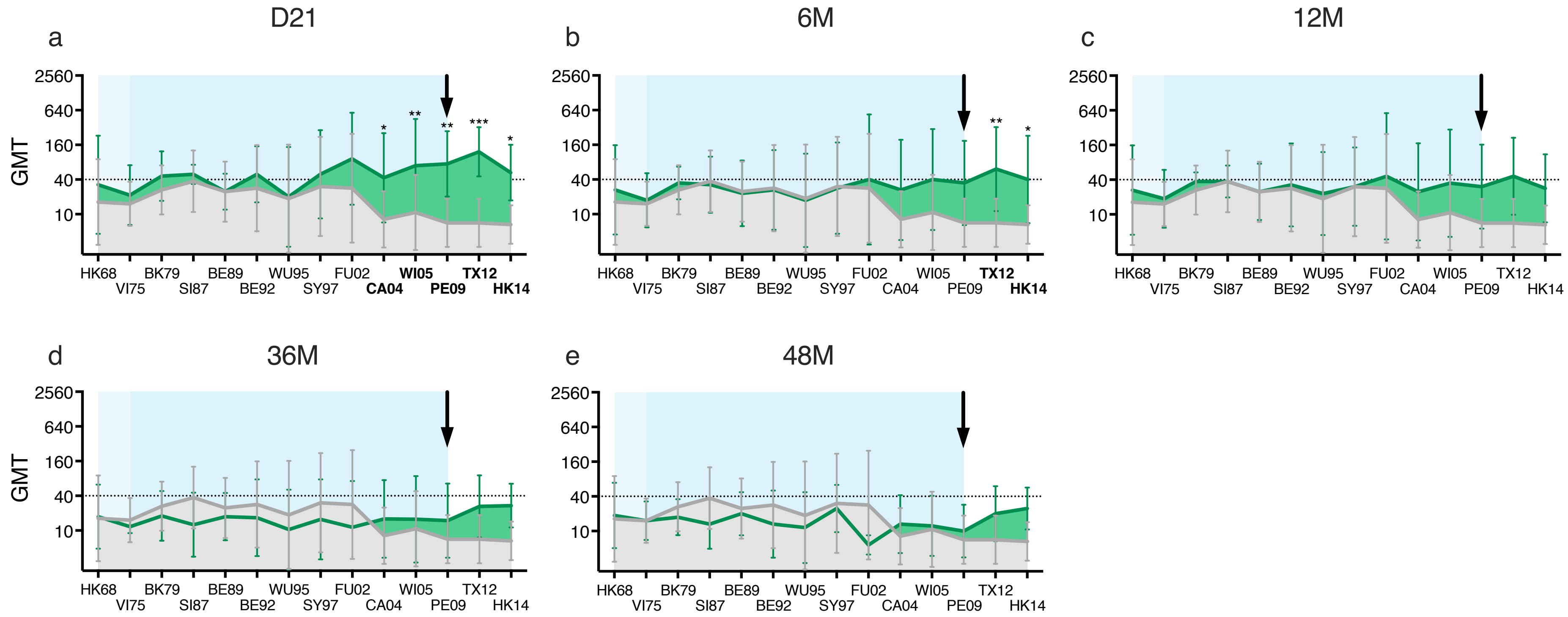

Selected subjects (n=5) from the 2010 IIV group provided blood samples for a long-term evaluation of antibody responses. The figures show geometric mean HI titres (GMTs) against 14 antigenically distinct influenza A/H3N2 viruses. Error bars represent the 95% confidence intervals of the GMTs. Grey pre-IIV titres Day 0 (D0, post-IIV titres in green at time-points day 21 (D21) (**a**), 6 months (6M) (**b**), 12 months (12M) (**c**), 36 months (36M) (**d**) and 48 months (48M) (**e**). The black arrow indicates the vaccination virus. The period of viral exposure is highlighted by a light blue background. Pre and post IIV HI titres were compared using non-parametric repeated measure Friedman test with Dunn's multiple comparison correction. *P <0.05, **P <0.01, ***P =<0.001.

Supplementary Figure 5: The impact of age and previous vaccination on the breadth of heamagglutination inhibition (HI) A/H3N2-specific antibody responses.

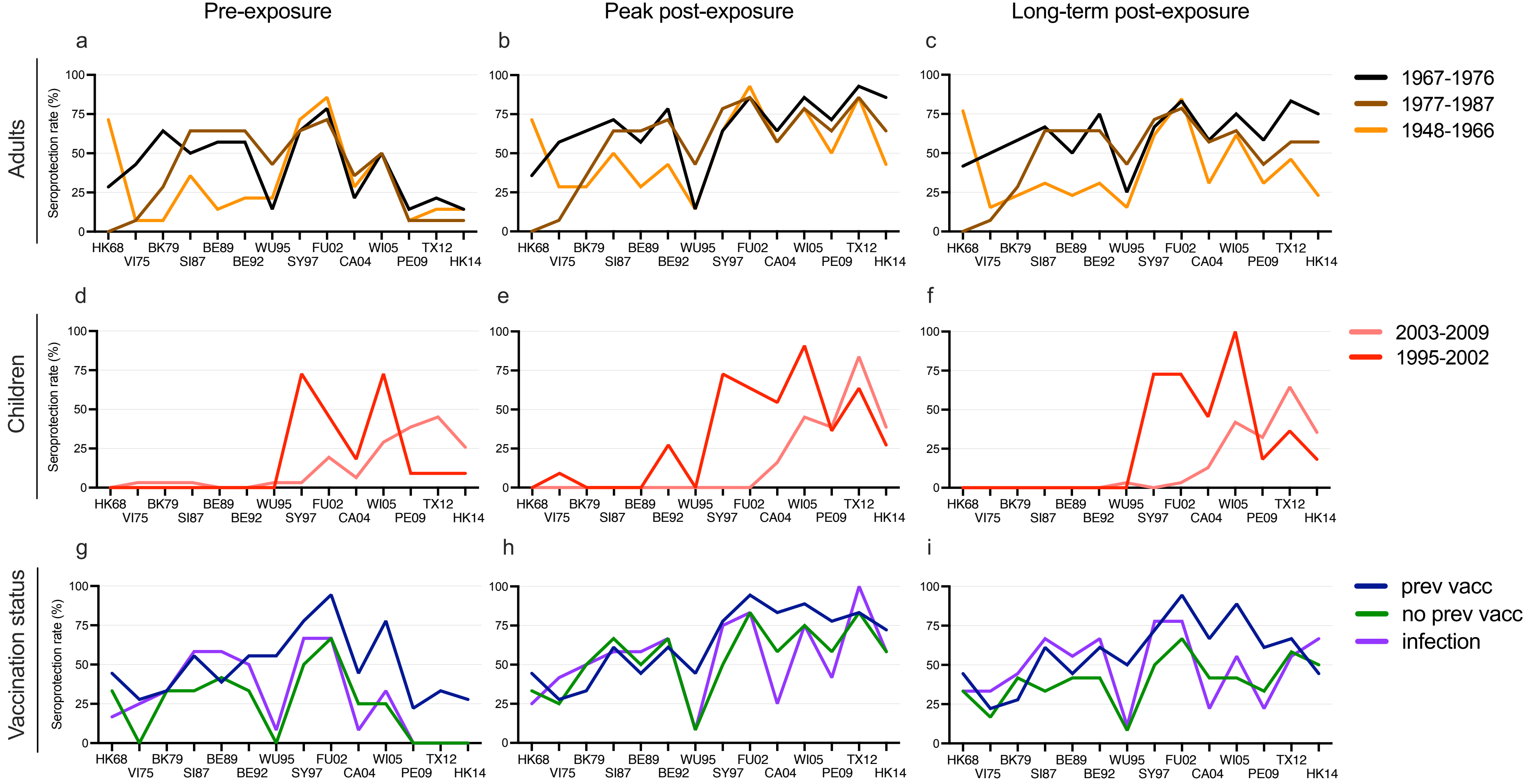

The seropositivity (percentages of individuals with HI titres ≥40) against the different influenza A/H3N2 viruses pre-exposure (pre-vaccination or pre-infection) (**a, d, g**), peak post-exposure (day 21/28 post-vaccination or 6 months post-infection) (**b, e, h**), and long-term post-exposure (6 months post-vaccination or 18 months post-infection) (**c, f, i**). Subjects were stratified by their birth year (**a-f**) or vaccination status (**g-i).** Adults were divided into 3 groups: born 1977-1987 (H1/H3 primed), 1967-1976 (H3 primed) and 1948-1966 (H1/H2 primed) (**a-c**) and children were divided into two groups: born 2003-2009 and 1995-2002 (both H1/H3 primed) (**d-f**). Adults were divided by previous vaccination history and compared to the infection group (**g-i**).

Supplementary Figure 6: Comparison of turkey and guinea pig blood in the haemagglutination inhibition (HI) assay against A/Hong Kong/4801/2014 (HK14)

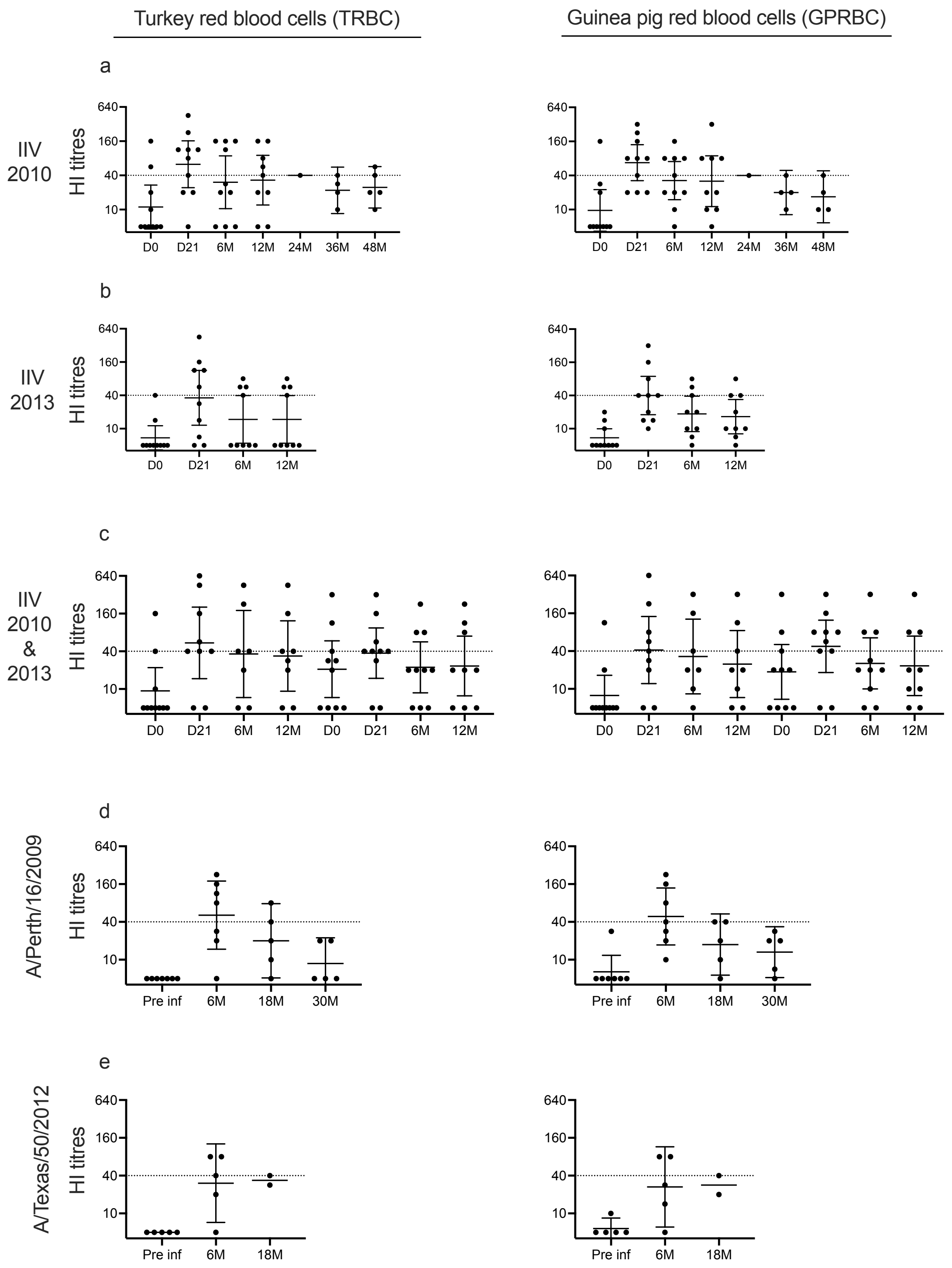

HI antibodies against HK14 were tested using turkey red blood cells (TRBC) or guinea pig red blood cells (GPRBC) in the different groups of adults, (**a**) inactivated influenza vaccine (IIV) in 2010 (single 2010 IIV group), (**b**) single 2013 IIV group, (**c**) double 2010 and 2013 IIV group, (**d**) infection group infected with A/Perth/16/2009 (H3N2), (**e**) infection group infected with A/Texas/50/2012 (H3N2). The procedures of HI assay using TRBC were described in the Methods. Briefly, serum samples were treated with receptor-destroying enzyme (Seiken, Japan) and pre-adsorbed with packed TRBC before serial dilution from 1/10 in duplicates and incubated with 4 hemagglutinating units of virus for 1 hour. The serum-virus mixture was further incubated with 0.5% (volume/volume) TRBC for 30 minutes and the HI titre was read as the reciprocal of the highest dilution of the test sample where complete inhibition of agglutination occurs. The procedures of HI assay using GPRBC were similar to TRBC, except that the serum-virus mixture was incubated with 0.7% GPRBC for 1 hour.
