# Supplementary material for: Seasonal influenza vaccination expands hemagglutinin-specific antibody breadth to older and future A/H3N2 viruses
Source: NPJ Vaccines. 2022 Jun 24;7:67. doi: 10.1038/s41541-022-00490-0 (PMC9232600; doi:10.1038/s41541-022-00490-0)
Supplement: Supplementary file 2 — REPORTING SUMMARY [file 41541_2022_490_MOESM2_ESM.pdf]

nature portfolio

Corresponding author(s): Mai-Chi Trieu

Last updated by author(s): Mar 22, 2022

Reporting Summary

Nature Portfolio wishes to improve the reproducibility of the work that we publish. This form provides structure for consistency and transparency in reporting. For further information on Nature Portfolio policies, see our Editorial Policies and the Editorial Policy Checklist.

Statistics

For all statistical analyses, confirm that the following items are present in the figure legend, table legend, main text, or Methods section.

n/a	Confirmed	
☐	☒	The exact sample size (*n*) for each experimental group/condition, given as a discrete number and unit of measurement
☐	☒	A statement on whether measurements were taken from distinct samples or whether the same sample was measured repeatedly
☐	☒	The statistical test(s) used AND whether they are one- or two-sided<br>*Only common tests should be described solely by name; describe more complex techniques in the Methods section.*
☐	☒	A description of all covariates tested
☐	☒	A description of any assumptions or corrections, such as tests of normality and adjustment for multiple comparisons
☐	☒	A full description of the statistical parameters including central tendency (e.g. means) or other basic estimates (e.g. regression coefficient) AND variation (e.g. standard deviation) or associated estimates of uncertainty (e.g. confidence intervals)
☒	☐	For null hypothesis testing, the test statistic (e.g. *F*, *t*, *r*) with confidence intervals, effect sizes, degrees of freedom and *P* value noted<br>*Give P values as exact values whenever suitable.*
☒	☐	For Bayesian analysis, information on the choice of priors and Markov chain Monte Carlo settings
☒	☐	For hierarchical and complex designs, identification of the appropriate level for tests and full reporting of outcomes
☒	☐	Estimates of effect sizes (e.g. Cohen's *d*, Pearson's *r*), indicating how they were calculated

Our web collection on statistics for biologists contains articles on many of the points above.

Software and code

Policy information about availability of computer code

Data collection	Data was collected on manual case report forms (CRFs).
Data analysis	All analysis were conducted in GraphPad Prism version 9.

For manuscripts utilizing custom algorithms or software that are central to the research but not yet described in published literature, software must be made available to editors and reviewers. We strongly encourage code deposition in a community repository (e.g. GitHub). See the Nature Portfolio guidelines for submitting code & software for further information.

Data

Policy information about availability of data

All manuscripts must include a data availability statement. This statement should provide the following information, where applicable:

- Accession codes, unique identifiers, or web links for publicly available datasets
- A description of any restrictions on data availability
- For clinical datasets or third party data, please ensure that the statement adheres to our policy

The datasets generated during and/or analysed during the current study are available from the corresponding authors on reasonable request. Supplementary Figures 2-4 show associated raw data.

Field-specific reporting

Please select the one below that is the best fit for your research. If you are not sure, read the appropriate sections before making your selection.

☒ Life sciences ☐ Behavioural & social sciences ☐ Ecological, evolutionary & environmental sciences

For a reference copy of the document with all sections, see nature.com/documents/nr-reporting-summary-flat.pdf

Life sciences study design

All studies must disclose on these points even when the disclosure is negative.

Sample size	The groups of vaccinated adults were chosen on the basis of the update of the H3N2-component in the seasonal vaccine, in order to compare between seasons. Adults who were sampled at all timepoints requested were prioritized. All non-vaccinated individuals who fulfilled the seroconversion criteria for a A/H3N2-virus were included, generating a total of 42 adults in the study. All children vaccinated in 2012 were chosen as study subjects, as well as most of the vaccinated children in 2013, excluding subjects with limited sample volume.
Data exclusions	Adults or children who did not deliver blood samples at the timepoints included in the study were excluded.
Replication	All serological assays were performed in duplicate with the mean of the titres used, as well as positive and negative controls.
Randomization	NA
Blinding	NA

Reporting for specific materials, systems and methods

We require information from authors about some types of materials, experimental systems and methods used in many studies. Here, indicate whether each material, system or method listed is relevant to your study. If you are not sure if a list item applies to your research, read the appropriate section before selecting a response.

Materials & experimental systems

n/a	Involved in the study	
☒	☐	Antibodies
☒	☐	Eukaryotic cell lines
☒	☐	Palaeontology and archaeology
☒	☐	Animals and other organisms
☐	☒	Human research participants
☐	☒	Clinical data
☒	☐	Dual use research of concern

Methods

n/a	Involved in the study	
☒	☐	ChIP-seq
☒	☐	Flow cytometry
☒	☐	MRI-based neuroimaging

Human research participants

Policy information about studies involving human research participants

Population characteristics	The adult research participants (n=42) were mainly healthy (2.4% with a high-risk condition), 76,2% female and median age 36 years old. The children (n=42) had a median age of 5 years and 47,6% were female.
Recruitment	Adults who received the pdmH1N1 vaccine were recruited to examine the immunogenicity of the vaccine and long-term follow-up was conducted with invegstigation of the immune response after seasonal influenza in the upcoming four influenza seasons. Primarily healthy children were recruited at the Department of Otorhinolaryngology, Haukeland University Hospital, scheduled for routine tonsillectomy and adenoidectomy to receive the live-attenuated influenza vaccine.
Ethics oversight	The studies were approved by the Regional Ethics Committee of Western Norway.

Note that full information on the approval of the study protocol must also be provided in the manuscript.

Clinical data

Policy information about clinical studies

All manuscripts should comply with the ICMJE guidelines for publication of clinical research and a completed CONSORT checklist must be included with all submissions.

Clinical trial registration	NIH Clinical trials.gov NCT01003288 (adults) and NCT01866540 (children).

Study protocol	Studies of Pandemic Influenza (H1N1) 2009 Vaccine in Bergen (H1N1VAC) and Immune Response To Intranasal Influenza Vaccination.
Data collection	The recruitment commenced in October 2009 for adult HCWs at Haukeland University Hospital, Bergen, Norway. For children recruitment was conducted in October 2012 and 2013 at Haukeland University Hospital, Bergen, Norway.
Outcomes	The primary endpoints of both trials were to evaluation of the systemic and local immune response to influenza vaccination. Furthermore, the capacity of the vaccines to elicit cross reactive and long-lasting immunity was evaluated. The immunogenicity was assessed through the induction of specific local and systemic antibody and cellular immune responses.
